# Extracellular vesicles from long COVID patients promote RUNX2-mediated cellular stress via dysregulated miR-204 and p53 pathway activation

**DOI:** 10.1186/s12964-025-02502-7

**Published:** 2025-11-26

**Authors:** Luca Dalle Carbonare, Arianna Minoia, Sharazed Zouari, Michele Braggio, Mattia Cominacini, Salvatore Calogero Gaglio, Francesca Cristiana Piritore, Pamela Lorenzi, Mirko Meneghel, Kevin Dervishi, Andrea Corsi, Anna Pedrinolla, Gaia Giuriato, Alessandra Fiore, Adriana Celesia, Laura Guerricchio, Massimo Venturelli, Federico Schena, Massimo Donadelli, Monica Mottes, Maria Grazia Romanelli, Massimiliano Perduca, Daniele Guardavaccaro, Ernesto Crisafulli, Donato Zipeto, Lucio Barile, Maria Teresa Valenti

**Affiliations:** 1 Department of Engineering for Innovation Medicine and School of Medicine in Sports and Exercise, University of Verona and Azienda Ospedaliera Universitaria Integrata of Verona, Verona, 37134 Italy; 2 Department of Neurosciences, Biomedicines and Movement Medicine, University of Verona, Verona, 37134 Italy; 3 Department of Experimental Medicine (DIMES), University of Genova, Genova, 16132 Italy; 4 Faculty of Biomedical Sciences, Università della Svizzera italiana, Lugano, CH-6900 Switzerland; 5 Cardiovascular Theranostics Group, Istituto Cardiocentro Ticino and Laboratories for Traslational Research Ente Ospedaliero Cantonale, Bellinzona, CH-6500 Switzerland; 6 Department of Biotechnology, University of Verona, Verona, 37134 Italy; 7 Department of Medicine, Respiratory Medicine Unit, University of Verona and Azienda Ospedaliera Universitaria Integrata of Verona, Verona, 37134 Italy

**Keywords:** Long covid, RUNX2, SESN, Hsa-miR-204-5p, Mesenchymal stem cells

## Abstract

**Background:**

Subjects with Long COVID, also known as post-acute sequelae of SARS-CoV-2 infection (PASC), experience a wide range of symptoms, including fatigue and respiratory disturbances, affecting their quality of life. Despite the increasing prevalence of Long COVID, the underlying pathogenic mechanisms remain poorly understood. Extracellular vesicles (EVs) are known to be involved in various processes, such as tissue repair and the transmission of viral particles. However, the specific characteristics and functional roles of EVs derived- from patients with Long COVID (LC-EVs) are poorly characterized.

**Methods:**

To uncover systemic mechanisms underlying Long COVID, we performed a comprehensive characterization of patient-derived extracellular vesicles (EVs) via Nanoparticle Tracking analysis (NTA), Atomic Force Microscopy (AFM), Transmission Electron Microscope (TEM) and flow cytometry. These EVs were applied to lung cells, Mesenchymal Stem Cell (MSCs), Human Umbilical Vein Endothelial Cells (HUVECs) and Aortic Smooth Muscle Cells (ASMCs), revealing stress responses through SESN1, SESN2, and p53 activation. We further assessed mitochondrial respiration to evaluate metabolic dysfunction, and conducted targeted transfection experiments to dissect the molecular pathways involved, shedding light on EV-driven cellular reprogramming.

**Results:**

Thus, we observed that Long COVID (LC) patients experienced breathlessness and leg discomfort during exertion. Our data highlighted that LC-EVs induce aberrant RUNX2 expression and activate the p53/p21 pathway in lung cells as well stress responses. Additionally, LC-EVs impair mitochondrial function and cellular adaptability under metabolic stress, reducing maximal respiration and ATP production at high cell densities. Protein interaction analysis showed RUNX2 involvement in key biological processes and post-transcriptional regulation by hsa-miR-204-5p was identified. Finally, LC-EVs also activated stress pathways and increased RUNX2, SESN, p53, and p21 levels in endothelial cells, aortic smooth muscle cells, and mesenchymal stem cells.

**Conclusions:**

In conclusions, these findings provide new insights into the role of extracellular vesicles in Long COVID, revealing their involvement in cellular stress and impaired mitochondrial function.

**Supplementary Information:**

The online version contains supplementary material available at 10.1186/s12964-025-02502-7.



## Introduction

Although more than five years have passed, severe acute respiratory syndrome coronavirus 2 (SARS-CoV-2) continues to circulate and poses a significant challenge to global health. Our understanding of the disease’s development is expanding, particularly about how it impacts multiple organs. COVID-19 effects may persist after the initial infection, causing damage to various organs [1]. These long-term effects may include both pulmonary and extra-pulmonary complications [1]. Therefore, subjects with Long COVID, also known as post-acute sequelae of SARS-CoV-2 infection (PASC), present symptoms such as chronic fatigue, shortness of breath, cognitive impairment, and a range of other issues impacting their quality of life [2–4]. It has been reported that between 31% and 69% of COVID-19 patients present long COVID symptoms [5]. Despite an increasing number of subjects presenting with long COVID symptoms, the pathogenic mechanisms causing Long COVID are still poorly understood.

Previous studies have shown a possible association between COVID-19 and the cargo of extracellular vesicles (EVs) [6]. Extracellular vesicles (EVs) are membrane-bound particles released by cells that facilitate intercellular communication by transporting proteins, lipids, and nucleic acids. EVs are broadly categorized into two main subtypes based on their biogenesis and size: exosomes (typically 30–150 nm in diameter), which originate from the inward budding of endosomal membranes and are released upon fusion of multivesicular bodies (MVBs) with the plasma membrane; and microvesicles (typically 100–1000 nm), which are formed by the outward budding and fission of the plasma membrane. These subtypes differ in origin, size, and molecular composition, contributing to their distinct biological roles [7]. EVs may be involved in immune modulation, tissue repair, and dissemination of viral particles. In the context of COVID-19, EVs have been found to carry viral RNA and proteins [8, 9], suggesting that they may play a role in symptom persistence. However, EVs’ specific characteristics and functional effects derived from Long COVID patients remain largely unexplored.

Thus, the hypothesis of this study was that extracellular vesicles (EVs) derived from Long COVID (LC-EVs) patients have distinct molecular and functional characteristics compared to those from healthy individuals and that these EVs may negatively affect cellular functionality, contributing to the pathogenic mechanisms of the disease. In addition, by examining key markers of cellular stress—such as SESN1, SESN2, and p53, which are involved in the response to metabolic and oxidative stress [10–13], we sought to elucidate potential mechanisms by which EVs contribute to the pathophysiology of Long COVID. These markers are part of a well-characterized p53-dependent stress response pathway [11, 13, 14]. In fact it has been demonstrated that p53 activation leads to the transcription of downstream targets including SESN1, SESN2, and p21 [13, 15]. Investigating this axis allowed us to explore whether LC-EVs might elicit stress responses in recipient cells through modulation of the p53 signaling pathway. Importantly, RUNX2 is a key transcription factor involved in the regulation of cellular differentiation, inflammatory responses and tissue remodeling [16–23]. Recently, it has been shown to modulate the expression of genes associated with chronic inflammation and fibrosis [24], processes closely linked to the long-term complications observed in COVID-19 patients [25]. Based on these findings, this study was designed as a hypothesis-driven investigation aimed at exploring whether cell-to-cell communication mediated by extracellular vesicles (EVs) can modulate cellular stress and thereby contribute to the persistence of Long COVID symptoms.

Thus, we investigated these aspects by characterizing LC-EVs isolated from patients and comparing them with those from healthy subjects. We used nanoparticle tracking analysis (NTA), transmission electron microscope (TEM) and atomic force microscopy (AFM) to assess the quantity, size, and morphology of these EVs. Furthermore, we investigated the impact of LC-EVson various cellular populations, including lung cells, mesenchymal stem cells (MSCs), endothelial cells (HUVEC) and aortic smooth muscle cells (ASMC).

Our findings suggest that LC-EVs carry distinct molecular cargos that may modulate cellular functions differently than EVs from healthy individuals. Understanding these mechanisms may provide new insights into therapeutic targets and strategies to alleviate the symptoms experienced by patients with Long COVID.

## Methods

### Aim, Design, and setting of the study

The aim of this study was to investigate the role of extracellular vesicles (EVs) in the pathogenesis of Long COVID (LC), focusing on their molecular composition and their functional effects on multiple cell types potentially involved in LC. We hypothesized that LC-EVs carry bioactive molecules associated with cellular stress contributing to the persistence of symptoms such as fatigue and breathlessness.

This was a comparative, in vitro experimental study involving EVs isolated from peripheral blood samples of 32 participants: 16 individuals diagnosed with Long COVID and 16 age- and sex-matched healthy controls EVs were characterized using nanoparticle tracking analysis (NTA), atomic force microscopy (AFM), transmission electron microscope (TEM) and flow cytometry to assess size, morphology and surface marker expression.

Functional assays were conducted by exposing various human cell lines—including lung epithelial cells (A549), primary human lung fibroblast (IMR90), human umbilical vein endothelial cells (HUVECs), aortic smooth muscle cells (ASMCs) and mesenchymal stem cells (MSCs)—to patient-derived EVs. We measured activation of stress-related pathways (SESN1/2, p53, p21), alterations in RUNX2 expression, and mitochondrial respiration capacity under metabolic stress. Transfection experiments were also employed to explore post-transcriptional regulatory mechanisms, particularly involving miR-204.

### Patients

Samples from 16 long COVID patients (8 males and 8 females) and 16 healthy (8 male and 8 female) were analyzed to evaluate the role of EVs. The healthy subjects (controls) had previously been infected with SARS-CoV-2 and had fully recovered, exhibiting no acute symptoms and no evidence of long COVID—defined, according to WHO criteria, as new or persistent symptoms lasting three months or more post-infection. Inclusion and exclusion criteria are described elsewhere (respicovid Crisafulli [26]): briefly, we evaluated outpatients previously hospitalized for SARS-CoV-2 infection. Male and female long COVID patients were BMI and age matched. All outpatients met the World Health Organization (WHO) criteria for the definition of long COVID (https://www.ncbi.nlm.nih.gov/books/NBK605675/). Each participant gave written informed consent. All subjects underwent a comprehensive clinical evaluation and a medical history interview to exclude any comorbidities. Written informed consent was obtained from all participants, and the study was approved by the Ethics Committee of the Azienda Ospedaliera Universitaria Integrata of Verona, Italy (no. 2785CESC). The study design and procedures adhered to the principles of the Declaration of Helsinki.

### Six-Minute walk test (6MWT)

The 6MWT was conducted on a straight 30 m rout. The start and endpoints were indicated by two Skittles, and the long COVID patients were instructed to walk as far as possible for six minutes. The protocol followed the American Thoracic Society (ATS) [27] guidelines. The distance walked (meters) was recorded. Predicted values were calculated via the equation by Enright et al. [28]. Heart rate (bpm) and pulse oximetry (sPO2) were monitored at rest and every minute (Pulsox-5, Konica Minolta Inc). Basal, peak and first-minute recovery blood pressure were also measured. 6MWT reference values for healthy subjects, obtained post-pandemic by De Soomer et al.show that healthy individuals perceive significantly less effort in terms of both dyspnea and muscle fatigue [29]. Taking these normative data into account, we did not perform the 6MWT in the control group.

### Pulmonary function tests

Lung function tests were conducted in accordance with international guidelines [30] and were expressed as percentages of predicted values (Official Statement of the European Respiratory Society [31, 32]). Measurements were obtained using a flow-sensing spirometer (Jaeger MasterScreen PFT System) connected to a computer for data analysis. The parameters recorded included forced vital capacity (FVC), forced expiratory volume in the first second (FEV1), and total lung capacity (TLC). Diffusing capacity (DLCO) and transfer coefficient (KCO) were assessed using the single breath method.

### Extracellular vesicles (EVs) isolation from human blood serum

Extracellular vesicles (EVs) were isolated using the qEVoriginal-70 nm Gen 2 column (Izon Science Limited ©), as previously reported [33]. Specifically, fractions collected between 0 and 2 mL were selected to reduce contamination from serum proteins [34]. The detection of proteins using Amido Black staining showed no traces of albumin in the selected EV isolation method. (Figure S1). In addition, the efficiency of this purification step was verified by Bradford colorimetric assay. Notably, the EV-containing fractions did not exhibit any reaction with the Bradford reagent (Figure S2), and no color change was observed. In contrast, fractions collected between 3.2 and 8 mL displayed a strong blue coloration, confirming the presence of serum proteins (Figure S2). These results indicate that the biological effects observed in downstream experiments can be solely attributed to the proteins and molecular components contained within the extracellular vesicles, which were successfully purified from serum protein contamination.

In addition, Western blot analysis was performed to assess the presence of standard EV markers (Figure S3). EV samples were prepared using two distinct protocols depending on the markers to be analyzed. For the detection of TSG101, Syntenin-1, ALIX, and Calnexin, EVs were boiled for 10 min at 95 °C in 6× Laemmli SDS sample buffer (VWR International, Dietikon). For the analysis of CD63 and CD81, EVs were instead incubated in 6× Laemmli sample buffer without SDS. Protein separation was performed on 4–20% Mini-PROTEAN^®^ TGX™ precast gels (Bio-Rad Europe), followed by transfer onto PVDF membranes using a semi-dry transfer system (Bio-Rad). Membranes were incubated with the following primary antibodies: anti-TSG101 (1:1000, Abcam, ab125011), anti-Syntenin-1 (1:1000, Abcam, ab19903), anti-ALIX (1:1000, Abcam, ab186429), anti-Calnexin (1:2000, Abcam, ab133615), anti-CD63 (1:1000, Invitrogen, 10628D), and anti-CD81 (1:1000, BD Biosciences, 555675). Thus, treatment of A549 cells with the later fractions (protein-rich fractions) of EVs isolationfrom either control or Long COVID samples did not induce significant changes in RUNX2 expression (Figure S4).

To minimize intra-group variability and obtain representative profiles, EVs were isolated from pooled plasma samples within each group (Long COVID patients and healthy controls), and the pooled samples were used for all downstream analyses, including NTA, TEM and flow cytometry.

### Nano tracking analysis (NTA)

NTA is based on the dynamic light scattering (DLS) phenomenon where the diffusion coefficient of each tracked particle is calculated in an aqueous environment by the Stokes–Einstein equation [35]. Thus, NTA was performed by using Malvern NanoSight NS300 instrument (Worcestershire, UK). NTA was also used to estimate EVs concentration before starting subsequent experiments.

### Atomic force microscopy (AFM)

AFM was performed using an NT-MDT Solver Pro atomic force microscope (Moscow, Russia) with NT-MDT NSG01 golden-coated silicon tip in semi-contact mode, applying a scanning frequency of 1 Hz in order to produce optimized AFM images. Furthermore, the microscope was calibrated by a calibration grating (TGQ1 from NT-MDT) to avoid nonlinearity and hysteresis in the measurements. EVs were first loaded on a bracket covered by an inert mica surface, and after 30 min of solvent evaporation, the analysis was performed. Finally, images were processed with the Scanning Probe Image Processor (SPIP™) program (*Friis Jan*,* J. Scanning Probe Image Processor 4.8)* [36].

### Tem analysis

One drop of the sample (about 25 µl) was placed on a 400 mesh holey film grid; after staining with 2% uranyl acetate (for 1 min) the sample was observed with a Tecnai G2 (FEI) transmission electron microscope operating at 100 kV. At least 20 images for each condition were captured with a Veleta (Olympus Soft Imaging System) digital camera.

### Flow cytometry and analysis

EVs samples were subjected to surface epitopes bead-based flow cytometry analysis (MACSPlex Exosome kit Miltenyi Biotec, Bergisch Gladbach, Germany) according to the manufacturer protocol. In brief, 4.62 × 10^7^ EVs from each condition (Control- and LC-EVs) were diluted in MACSPlex buffer to a total volume of 120 µl and incubated with MACSPlex exosome capture beads overnight. APC-labeled CD6, CD63 and CD81 antibodies were used for counterstaining. APC-A values were acquired with a BD LSRFortessa X-20 cytometer (BD biosciences, Heidelberg, Germany) and the FlowJo software (Ashland, Oregon, United States) was used to analyze data. For each sample, the median signal intensity of the signals detected for the CD9, CD63 and CD81 capture beads was calculated, and their geometrical mean was used as the normalization factor for each sample.

### Cell culture

The A549 cell line was obtained from the American Type Culture Collection (CCL-185-ATCC ATCC, Philadelphia, PA, United States). Cells were maintained at 70% of confluency in DMEM (Cat. Number 11965092, Gibco, Thermo Fisher Scientific, Waltham, MA, USA) supplemented with 10% FBS (Cat.Number 10270106, Gibco, Thermo Fisher Scientific, Waltham, MA, USA) and 1% penicillin-streptomycin (PSA) (Cat.Number 17-745E, Lonza, Basel, Switzerland). IMR90 cell line was obtained from the American Type Culture Collection (CCL-186-ATCC, Philadelphia, PA, United States). Cells were maintained at 70% of confluency in DMEM, supplemented with 10% FBS and 1% penicillin-streptomycin. Human Aortic Smooth Muscle Cells (HAOSMCs) **(**HAOSMCs) (Cat. Number C-12533, PromoCell, Heidelberg, Germany**)** were maintained at 70% of confluency in Smooth Muscle Cell Growth Medium 2 (Cat.Number C-22062, PromoCell, GmbH Sickingenstr. 63/65 69126 Heidelberg, Germany. Cat.No. C-22062), enriched with the proprietary SupplementMix (Cat.Number C-39267, PromoCell, GmbH Sickingenstr. 63/65 69126 Heidelberg, Germany Cat.No. C-39267) containing FCS, Epidermal Growth Factor, Basic Fibroblast Growth Factor and Insulin; mediums were enriched with 1% PSA. Human bone marrow-derived mesenchymal stem cells (hBM-MSCs) (Cat.Number C-12974, PromoCell, Heidelberg, Germany) were maintained at 70% of confluency in the MesenPRO RS Basal Medium (12747-010, Gibco, Life Technologies Corporation, 3175 Staley Rd., Grand Island, NE, USA) supplemented with 10% supplement mix and 1% penicillin-streptomycin-amphotericin (Cat.Number 17-745E, Lonza, Basel, Switzerland). HUVEC cells were obtained from Promocell (Cat. No: C-12200, Heidelberg, Germany) and maintained at 70% of confluency in presence of M200 medium (Cat.Number M200500, Thermo Fisher Scientific, Waltham, MA, USA), supplemented with 10% low serum growth supplement (LSGS) (Cat. Number S00310 Thermo Fisher Scientific, Waltham, MA, USA) and 1% penicillin-streptomycin amphotericin.

All cells were cultured in a humidified CO_2_ incubator at 37 °C. Cells at 80% confluence were treated with EVs extracted from serum pools belonging to long covid patients or controls. EVs were added directly to fresh medium, with a final concentration of 9.8 × 10^8^ EVs/mL. Treatment with the conditioned medium lasted for 18 h before collection. The cells were collected using 1% trypsin (Cat. Number 59427 C, Merck, Rahway, New Jersey (USA). The EV concentration and exposure time (9.8 × 10⁸ EVs/mL for 18 h) were selected based on preliminary experiments conducted in our laboratory confirmed that this dose did not induce cytotoxic effects and ensured sufficient EV uptake in cells.

### Cellular staining

A549, HUVEC, HAOSMC, and MSCs cells were separately seeded onto chamber slides, each in their respective basal media. Cells were then stained with a vital fluorescent dye, the Vybrant Cell Labeling Solution (Invitrogen, Waltham, MA, USA), using 3,3′-dioctadecyloxacarbocyanine perchlorates (DiO), which emits green fluorescence at 501 nm. The cell nuclei were stained with Hoechst dye (Thermo Fisher Corporation, Waltham, MA, USA). EVs were stained with 1,1′-dioctadecyl-3,3,3′,3′-tetramethylindocarbocyanine perchlorate (DiI), a red fluorescent dye with emission at 565 nm, and added to the cells for conditioning.

Following an 18-hour incubation, cells were fixed with 4% paraformaldehyde (PFA) and visualized using the EVOS M5000™ Core Imaging System (Invitrogen, ThermoFisher Scientific, Waltham, MA, USA).

### Immunofluorescence

Immunofluorescence analysis was conducted as previously described by Deiana et al. [37] on A549 cells conditioned with EVs. Briefly, cells were fixed with 4% paraformaldehyde (PFA), permeabilized with 0.5% Triton X-100, and processed as previously reported [37]. The primary antibodies CK19 (Ref: PA0468, Leica, Wetzlar, Hesse) and Vimentin (Ref: PA0640, Leica, Wetzlar, Hesse) were applied as specified in the respective datasheets and incubated overnight at 4 °C.

The slides were then incubated with Alexa-Fluor 488-conjugated anti-goat secondary antibodies in mice (Cat. Number A25618, Invitrogen, ThermoFisher Scientific, Waltham, MA, USA), diluted 1:300 as recommended by the datasheet. Nuclear staining was performed using ProLong™ Gold Antifade Mountant with DAPI (Cat. Number P36941, Invitrogen, ThermoFisher Scientific, Waltham, MA, USA). Negative controls were performed by using only secondary antibodies **(Figure S5)**. Images were captured using the EVOS M5000™ Core Imaging System (Invitrogen, ThermoFisher Scientific, Waltham, MA, USA).

### Cell transfections

The RUNX2 gene was cloned into the pcDNA3 expression vector, as we previously reported [37]. Briefly, the amplified RUNX2 gene sequence (NM_001024630.4) was initially inserted into the pCR™ 2.1 vector (Thermo Fischer Scientific, Waltham, MA, USA), then excised using *EcoRV/XhoI* digestion and cloned into the pcDNA3-Flag-HA vector (Addgene plasmid #10792, Watertown, MA, USA) [37]. Transfection of the RUNX2-expressing plasmid was carried out using RUNX2 Lenti ORF particles from Origene Technologies (Rockville, MD, USA), following the manufacturer’s instructions. A549 cells were grown to 70% confluence and subsequently transfected with 10 µg/mL RUNX2-expressing pcDNA3 vector, using Lipofectamine 3000 (Thermo Fisher Scientific, Waltham, MA, USA; reference: L3000-008), as we previously reported [38].

To evaluate the increase in hsa-miR-204-5p expression, A549 were transfected with a mirVana™ hsa-miR-204-5p mimic (Catalog number: 4464066 ID: MC11116, Invitrogen by Thermo Fisher Scientific) at different concentrations (5 nM, 10 nM, 15 nM, 20 nM, 25 nM) or silencer-negative control siRNA (Cat#: AM14611, Ambion by Thermo Fisher Scientific, Waltham, MA, USA). The A549 were plated at a density of 3.5 × 10^5^ cells in T25 flasks and cultured in the presence of DMEM. At a cell confluency of 70%, transfection was carried out using Lipofectamine 3000 (L3000-008, Invitrogen by Thermo Fisher Scientific Baltics UAB, Vilnius, Lithuania). Cells were incubated for 24 h hours with the transfection complex; after incubation cells were collected and samples stored until use.

For RUNX2 silencer, transfection was carried out according to the manufacturer’s instructions using Lipofectamine 3000. A549 were transfected with Silencer SelectPre-designed siRNA RUNX2 (REF: 4392420, siRNA ID: s2456 Invitrogen by Thermo Fisher Scientific) and silencer-negative control siRNA. The cells were transfected for 24 h and treated with Long Covid EVs for 18 h. After transfection and treatment, cells were harvested using trypsin 1%, and the cell pellet was processed for protein extraction.

### Western blotting

Protein concentration was determined using the BCA Protein Assay Kit (Cat. Number eMP014500, Quantum Protein, Bicinchoninic Protein Assay, Euroclone S.p.A. Società a Socio Unico Via Figino, 20/22 20016 Pero, MI) and measured with a VICTOR Microplate Reader (PerkinElmer, Waltham X3-2030-0030, MA, USA). Subsequently, protein samples were normalized with Pierce™ RIPA buffer (Cat.Number: 78501 Thermo Fisher Corporation, Waltham, MA, USA) 1X and Loading Buffer. After incubating at 99 °C for 7 min, samples were loaded onto precast Mini-PROTEAN^®^ TGX gels (Bio-Rad, 2000 Alfred Nobel Dr, Hercules, CA, United States, California) for SDS-PAGE. SDS-PAGE was conducted in Running Buffer containing Tris (Cat.Number T1503, Merck, Rahway, New Jersey (USA)), Glycine (Cat.Number ECB2072, Euroclone S.p.A. Società a Socio Unico Via Figino, 20/22 20016 Pero, MI) and SDS (Cat. Number 11667289001, Merck, Rahway, New Jersey (USA)at 125 V for 15 min, then at 145 V for 1 h. Proteins were transferred to a PVDF membrane (Cat.Number: 88518 Invitrogen- Thermo Fisher Corporation, Waltham, MA, USA), activated with ethyl alcohol and water, and immersed in Transfer Buffer 1 × (1 L of 10X solution, 30.3 g of Tris (Cat.Number T1503, Merck, Rahway, New Jersey (USA)), 144.0 g of Glycine (Cat.Number ECB2072, Euroclone S.p.A. Società a Socio Unico Via Figino, 20/22 20016 Pero, MI, Italy) for 15 min. The transfer, using a Mini-PROTEAN^®^ Tetra Cell apparatus (Bio-Rad, 2000 Alfred Nobel Dr, Hercules, CA, United States, California) at 100 V in ice, lasted 1 h. After, the membrane underwent Amido Black staining (Cat. Number A8181- Sigma-Aldrich-St.Louis, Missouri, USA) and verification, with bands observed after destaining in MilliQ water for 10 min (Cat. Number: P7170- Sigma-Aldrich-St.Louis, Missouri, USA) the membrane was blocked with 5% fat-dry milk (Cat. Number 42590.02, SERVA Electrophoresis GmbH, Carl-Benz-Str., 69115 Heidelber) in TBS-Tween (Cat.Number 1706435, Bio-Rad, 2000 Alfred Nobel Dr, Hercules, CA, United States, California) for 1 h, followed by overnight incubation with the primary antibody at 4 °C. Simultaneously, the following antibodies were used: RUNX2 1:1000 55–62 kDa, (Cat.Number 8486 Cell Signaling- 3 Trask Lane Danvers, MA 01923), p53 1:1000 53KDa.(Cat.Number: FNab06083, FineTest), p21 1:1000 21KDa (Cat. Number: GTX629543, GeneTex), SESN1 1:500 57KDa, (Cat.Number: PA5-98142- Invitrogen Thermo Fisher Corporation, Waltham, MA, USA), SESN2 1:1000 54KDa (Cat Number: ab-178518 Abcam), Phospho-p53 (Ser15) (16G8) (Cat. N°9286, Cell signaling technologies, Danvers, Massachusetts, USA). β-actin 1:10000 42–55 kDa (Cat. Number: MA1-140-Invitrogen Thermo Fisher Corporation, Waltham, MA, USA) served as a housekeeping protein.

### RNA extraction and reverse transcription

Total RNA was extracted using the RNeasy^®^ protect mini kit (Qiagen, Hilden, Germany) following the manufacturer’s protocol. The quantity and quality of RNA samples were assessed using the ‘Qubit™ RNA HS assay kit’ (Invitrogen, Thermo Fisher Scientific, Waltham, MA, USA) and a Qubit 3 Fluorometer (Invitrogen, Thermo Fisher Scientific, Waltham, MA, USA -REF Q3321). RNA quality was assessed by spectrophotometric analysis by using NanoDrop ND-100 spectrophotometer (ThermoFisher Scientific, Waltham, MA, USA). Both A260/280 and A260/230 ratios were measured to evaluate purity, and our samples showed values close to ~ 2.0 (A260/280) and 2.0–2.2 (A260/230), which are indicative of good RNA quality.

For total RNA, reverse transcription was performed with the first-strand cDNA synthesis kit (GE, Healthcare Bio-sciences AB, Uppsala) following the manufacturer’s protocol. The thermocycler steps were performed using a GeneExplorerTM Thermal Cycler.

### Gene expression analysis-Real-time PCR

The Real-Time PCR was performed using the TaqMan Universal PCR Master Mix (Thermo Fisher Scientific, Waltham, MA, USA) with the following commercially pre-designed probes (Table S1) and the iTAQ Universal SybrGreen SuperMix (Bio-Rad, 2000 Alfred Nobel Dr, Hercules, CA, United States, California) with the primers reported (Table S2).

Next, The MicroAmp Optical 96-Well Reaction Plate (applied biosystems, Thermo Fisher Scientific, Waltham, MA, USA) was used to load the 19 µL of PCR mix into designed wells. Next, 1 µL (equivalent to 10 ng, after concentration adjustment) of cDNA was added. Three copies of each sample were loaded. Following a brief centrifugation, the plate was covered and sealed using the proper membrane (Opti-seal, AB analitica). For this procedure, the LineGene 9620 Real-Time PCR System (Aurogene, Hangzou Bioer Technology) was used. The PCR stage was repeated for 45 cycles following the different steps and lengths using the Taqman Master Mix or the SybrGreen SuperMix as previously reported [39]. At least three independents’ analyses were performed, and each analysis was performed in triplicate with Ct values averaged.

### MiRNA 204-5p- EVs

MiRNAs were extracted from EVs using the qEV RNA Extraction Kit (Izon Science Limited) according to the manufacturer’s instructions. The concentration and purity of the extracted miRNAs were assessed using a Nanodrop ND-1000 (Thermo Scientific). Specific miRNA 204-5p was reverse-transcribed into cDNA using the TaqMan™ MicroRNA Reverse Transcription Kit (4366596, Applied Biosystems by Thermo Fisher Scientific, Baltics UAB, Vilnius, Lithuania) with the miRNA-specific primer, following the manufacturer’s protocol. PCR amplification was performed in a total volume of 20 µL, including 2 µL of cDNA and TaqMan Universal PCR Master Mix. TaqMan miRNA-specific probes for hsa-miR-204-5p-5p (000508, 20X, FAM; Thermo Fisher Scientific, Life Technologies Corporation, Carlsbad, CA, USA) were obtained from Applied Biosystems (Thermo Fisher Scientific, Life Technologies Corporation, Carlsbad, CA, USA). U6 snRNA (001973, 20X, FAM; Thermo Fisher Scientific, Life Technologies Corporation, Carlsbad, CA, USA) was used as housekeeping control, showing stable expression across samples. The PCR was conducted for 45 cycles. Results were normalized to the housekeeping U6 snRNA, and relative fold expression differences were calculated using the ΔΔCt method as previously reported [40].

### High-resolution mitochondrial respirometry

A549 cells were plated in a T75 flask. Cells were detached with 0.05% trypsin and counted by trypan blue exclusion. After centrifugation at 200 gmax for 5 min at 25 °C, medium was removed, and cells were suspended in PBS and aliquoted for respirometry. A total of 2 × 10^6^ A549 cells were resuspended directly in the 2 mL Oroboros Oxygraph-2k (Oroboros Instruments, Corp., Innsbruck, Austria) chambers, filled with Mir05 medium (0.5 mM EGTA, 3 mM MgCl2·6 H2O, 60 mM lactobionic acid, 20 mM taurine, 10 mM KH2PO4, 20 mM HEPES, 110 mM sucrose, 1 g/L BSA). The published substrate-uncoupler-inhibitor titration (SUIT)−008 O2 ce-pce D025 was used to measure O_2_ Flux in pmol⋅s^–1^⋅2 million^–1^ cells. The assay was performed at 37 °C. Catalase was added to create MiR06, with a final concentration of 280 U/mL to induce hyperoxia. After measuring endogenous whole-cell respiration (ROUTINE state), cells were permeabilized by the addition of digitonin. In particular, we identified the optimal concentration of digitonin for cell permeabilization by performing titration experiments determining that a final concentration of 50 µg/mL was the most effective. Thus, substrates and inhibitors were added in the following order and final concentrations: pyruvate (5 mM) and malate (2 mM), ADP (2.5 mM), glutamate (10 mM), succinate (10 mM), carbonyl cyanide m-chlorophenylhydrazone (CCCP, multiple 0.5 μm titrations), rotenone (0.5 µM), and antimycin A (2.5 µM). Residual oxygen consumption was subtracted from the oxygen flux as a baseline for all respiratory states. Data analysis was performed using DatLab 5.2 software (Oroboros, Paar, Graz, Austria). Chemicals were purchased from Sigma Aldrich/Merck Life Science. This titration method was completed within 90 min.

### Seahorse

In order to assess mitochondrial function, A549 cells were seeded at the density of 1.5 × 10^4^ cells/well or 2 × 10^4^ cells/well and IMR90 cells were seeded at density of 1 × 10^4^ cells/well in a V7 XFe24-well cell-culture microplate and contextually treated with Control (CTRL) or Long Covid EVs (LC EVs). After 18 h, the media was changed with XF-RPMI medium (Seahorse Bioscience, cat. No. 103576-100) supplemented with 10 mM glucose (Agilent Technologies, Milan, Italy 103577-100), 1 mM Sodium Pyruvate (Agilent Technologies, Milan, Italy 103578-100), and 2 mM glutamine (Agilent Technologies, Milan, Italy 103579-100), pH 7.4, and the cells were incubated at 37 °C in a non-CO_2_ incubator for 1 h. Then, the Cell Mito Stress Test was performed by using the specific kit (Seahorse XF Cell Mito Stress Test Kit. 103015-100) in a Seahorse XFe24 Extracellular Flux Analyzer (Agilent Technologies, Milan, Italy), following manufacturer’s instructions. The oxygen consumption rate (OCR), as a readout of mitochondrial function, was measured at the baseline and after sequential addition of 1 mM oligomycin A (port A), 2 mM of carbonyl cyanide 4-(trifluoromethoxy) phenylhydrazone (FCCP, port B), and 1 mM each of Rotenone and Antimycin A (port C). Raw OCR data were normalized to the DNA content per well (µg DNA/well) that was quantified with the CyQUANT Cell proliferation assay kit (Thermo Fisher Scientific, cat. No. C35007), in accordance with manufacturer’s instructions. The OCR profiles are shown in Figure S6.

### Statistical analysis

The data are presented as mean ± standard deviation (SD) or mean ± standard error of the mean (SEM), as indicated. Statistical analyses included Student’s paired t-test for two-group comparisons and one-way or two-way analysis of variance (ANOVA) for comparisons across multiple groups. Post hoc multiple comparisons were conducted using Dunnett’s/Tukey’s test to determine specific group differences. Adjustments for multiple comparisons were applied to control the family-wise error rate, ensuring the overall type I error rate remained at α = 0.05. Although normality and homoscedasticity were not formally tested, the use of parametric methods is supported in small to moderate samples [41].

For the statistical analysis of qPCR data, we used the log2 fold change values calculated via the ΔΔCt method. Analyses were based at least on two (for analyses performed on Oroboros) or three independent experiments, each performed in triplicate. Statistical evaluations were conducted using SPSS for Windows, version 22.0 (SPSS Inc., Chicago, IL, USA), or GraphPad Prism (version 9), with a p-value ≤ 0.05 considered statistically significant.

## Results

### Long Covid patients

Sixteen patients were evaluated 4.5 ± 0.6 months after COVID19. Characteristics of long COVID patients are reported in Table 1. The severity of the initial infection required hospitalization in all patients. Ten of 16 needed O_2_ therapy and 2 were admitted into the intensive care unit. The duration of the hospitalization was 9.7 ± 6.2 days. All Long COVID patients consistently reported signs of fatigue, often described as overwhelming and persistent, interfering significantly with daily activities and quality of life. In particular, persisting symptoms included fatigue in seven patients; one patient reported resting dyspnea, and four experienced exercise-induced dyspnea. Additionally, three Long COVID patients reported a persistent cough. Spirometry parameters of subjects are reported in Table 2. The *6-minute walking distance* Test (6MWT) revealed a normal functional status in 15/16 patients, with only one male long COVID patient that covered a distance < 80% of the predicted value. Interestingly, at the modified CR10 Borg Scale [42] after the 6-minute walking, patients reported an exertional breathlessness rate of 2.5 ± 1.86 and a leg discomfort rate of 3.25 ± 1.94. Leg discomfort seemed to be more pronounced than perceived dyspnea (*p* < 0.05). Pulse oximetry values at rest (97.1 ± 1.1) and at peak exercise (96.5 ± 1.6) showed no significant differences (*p* = 0.120); however, 2 patients had a sPO2 < 95% after the 6MWT. Data of 6MWT are reported in Table 3.

**Table 1:** Characteristics of long COVID outpatients

ID	sex	Age (years)	Height (cm)	Weight (kg)	BMI (kg/m2)	LOS (days)	FUP (months)
1	M	57.5	174	97	32.0	6	4.0
4	M	63.8	170	74	26.0	11	4.0
9	M	65.1	170	81	28.0	9	4.0
11	M	65.7	164	72	26.8	14	4.0
17	M	61.7	182	83	25.1	11	4.0
21	M	62.7	182	81	24.5	2	4.0
29	M	57.4	171	71	24.3	3	4.0
34	M	55.3	174	88	29.1	3	4.0
48	F	55.5	172	62	21.0	6	5.0
49	F	59.6	165	64.5	23.7	9	5.5
50	F	61.0	149	86	38.7	11	4.0
54	F	56,9	160	98	38,3	17	4.0
56	F	55	164	59	21,9	3	4.0
63	F	60.0	155	66.5	27.7	14	4.0
68	F	55	159	67	26,5	11	4.0
74	F	64	150	70	31,1	25	4.0

Characteristics of long COVID outpatients

*LOS* Length of hospital stay for COVID19 infection, *FUP* Follow-up period after hospital discharge, *BMI* Body mass index

**Table 2:** Spirometry parameters of subjects

ID	FVC (l)	FVC%	FEV1 (l)	FEV1%	FEV1/FVC	TLC (l)	TLC%	DLCO_SB (ml/min/mmHg)	DLCO_SB%
1	5.09	121	3.88	116	76.22	6.84	100	26.78	94
4	4.41	115	2.87	95	64.99	4.72	96	17.64	96
9	4.68	125	3.43	117	72.31	6.69	103	26.44	103
11	2.73	80	2.11	79	77.16	4.42	73	18.22	77
17	4.44	97	3.49	98	78.7	6.78	91	26.59	88
21	5.22	115	4.29	121	81.94	7.51	101	30.69	102
29	4.85	120	4.03	126	83.19	6.65	101	23.11	84
34	5.22	123	4.29	126	82.06	7.13	104	24.42	85
48	4.62	140	3.9	138	84.32	5.84	105	21.65	84
49	3.91	135	3.23	132	82.65	5.52	108	21.13	90
50	2,21	104	1,70	97	77,15	3,30	82	20,01	104
54	4,04	147	3,39	146	84,04	5,50	115	19,27	85
56	3,24	109	2,81	111	86,70	5,03	100	15,78	66
63	2,72	112	2,30	112	84,30	4,09	92	16,78	80
68	3,60	132	2,67	116	74,36	4,90	104	20,75	92
74	2,52	119	2,08	119	82,32	3,58	87	14,14	73

*FVC* Forced vital capacity, *FEV1* First second forced expiratory volume, *FEV1/FVC* Tiffenau index, *TLC* Total lung capacity; *DLCO_SB* Single breath diffusing capacity of the lung for carbon monoxide

**Table 3:** 6-minute walking test (6MWT) of long COVID outpatients

ID	Distance (m)	%pred	BorgD rest	BorgL rest	BorgD peak	BorgL Peak	SpO2 rest	SpO2 peak	HRrest (bpm)	HRpeak (bpm)
1	414,00	75,4	0	0	2	5	98	95	79	118
4	500	94,8	0	0	8	8	98	97	59	126
9	495	97,3	0	0	1	1	98	95	94	116
11	472	99,2	0	0	2	4	98	98	60	83
17	548	89,4	0	0	4	4	97	96	80	108
21	669	109,4	0	0	0	1	97	97	70	68
29	699	122,2	0	0	1	1	97	97	80	121
34	709	123,1	0	0	2	3	97	96	89	153
48	600	105,8	0	0	3	4	97	98	54	97
49	505	96,6	0	0	3	3	97	95	83	121
50	687	159,9	0	0	3	1	94	94	66	118
54	448	99,3	0	3	4	5	96	97	64	68
56	600	106,6	0	0	3	4	97	98	70	97
63	447	89,8	0	0	1	1	97	97	86	118
68	563	106,0	0	0	1	3	99	100	76	110
74	420	92,5	0	0	3	4	96	94	75	89

%pred: percentage of the predicted distance value; BorgDrest and BorgDpeak,: points of the Borg scale of perceived dyspnea at rest and at the end of 6MWT; BorgLrest and BorgLpeak: points of the Borg scale of perceived leg fatigue at rest and at the end of 6MWT; SpO2: O2 pulse oximetry saturation

*HR* Heart rate

### EVs analyses from Control and Long COVID Samples

**Fig. 1:**
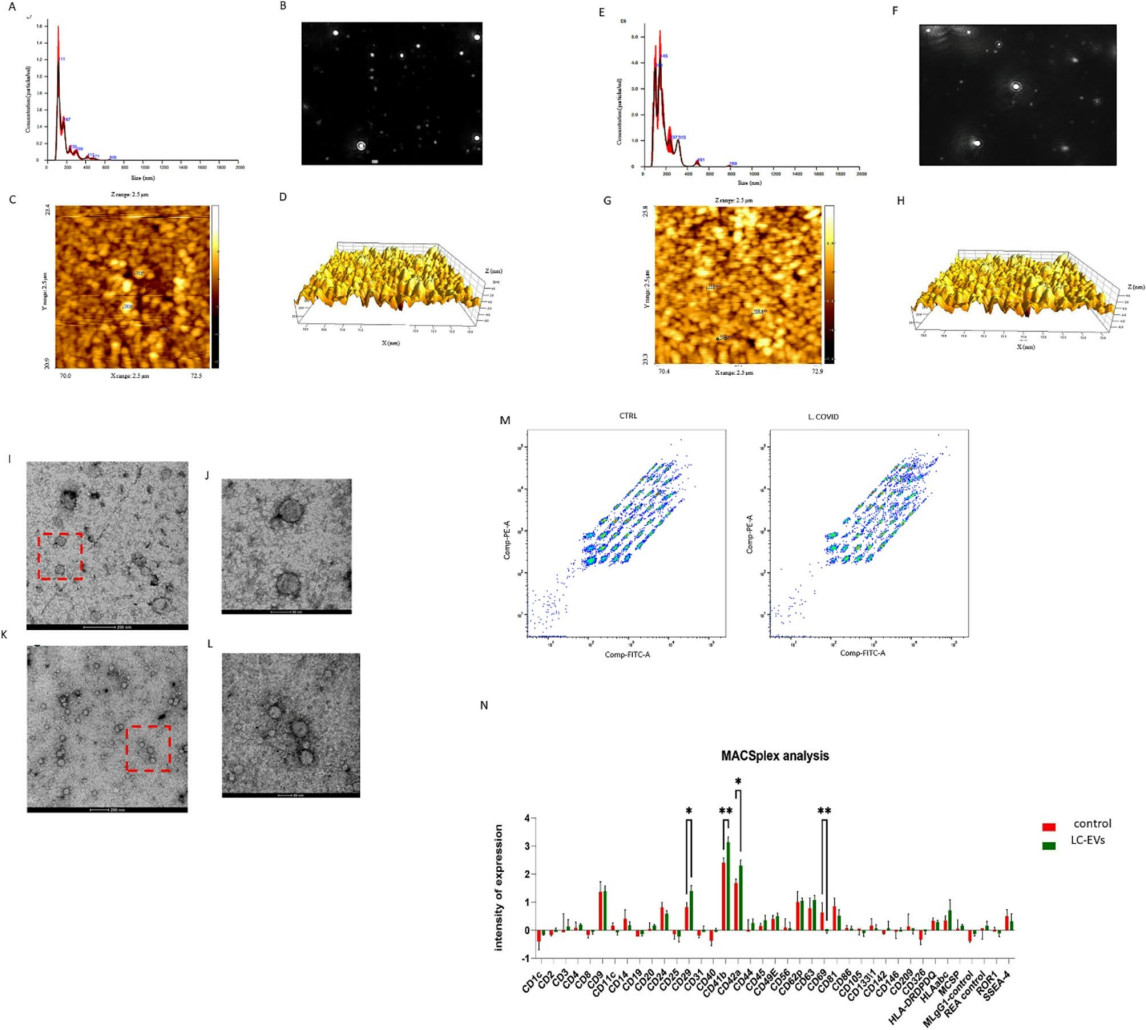
Characterization of EVS. **A** NTA size diagram of EVS from controls, **B** NTA camera capture of EVs light scattering from controls, **C** 2D and **D** 3D AFM analysis of EVs from controls. **E** NTA size diagram of LC-EVs, **F** NTA camera capture of EVs light scattering, **G** 2D and (**H**) 3D AFM analysis. **I** Representative TEM images of control EVs acquired at 200 nm resolution and 50 nm resolution (**J**), and of LC-EVs at 200 nm resolution (**K**) and 50 nm resolution (**L**). **M** Flow cytometry analyses: The dot plot represents fluorescence intensity data, with APC-A values indicating the expression levels of the analyzed markers. Diagonal clustering suggests a correlation between markers, potentially due to their co-expression on extracellular vesicles (EVs). The applied normalization factor, calculated as the geometric mean of CD9, CD63, and CD81 bead signals, ensures comparability between samples (**N**). MACSPlex analysis of extracellular vesicles (EVs) from Control (CTRL, green) and Long Covid (L.COVID, purple) samples. The bar graph represents the intensity of expression for various surface markers detected using bead-based flow cytometry. Three independent replicates were performed for both NTA and AFM analyses. Three replicates were carried out for flow cytometry experiments. (*p = 0.048; ** *p* = 0.0036)

EVs of both control and Long COVID subjects (Fig. 1A-N) were analyzed using Nanoparticle Tracking Analysis (NTA), Atomic Force Microscopy (AFM) and Transmission Electron Microscope (TEM). These techniques allowed us to accurately assess the size distribution and morphological characteristics of the EV populations, providing a comprehensive comparison between the two groups.

The differences observed (Table 4), both in the mean and mode values, are within the standard deviation range.

**Table 4:** Comparison between NTA output with AFM Diameter. Each measurement was performed in triplicate; for AFM analysis, a population of 30 particles was used to calculate AFM mean and mode

	NTA		AFM
Sample	Mean (nm)	Mode (nm)	Diameter (nm)
EVs (C)	171.9 (± 1.3)	107.8 (± 3.5)	128.5 (± 15.1)
EVs (LC)	176.1 (± 2.6)	126.9 (± 9).	137.1 (± 30.6)

TEM provided images of vesicles after dehydration and negative staining. The extra cellular vesicles appear predominantly spherical and the bilayer structure can be distinguished, as indicated by the higher contrast observed along the vesicle edges (Fig. 1I-L). Therefore, based on this analysis, there are no substantial differences in the size distribution between the two populations.

Moreover, the quantification of EVs did not reveal any significant differences between the Control and Long COVID groups (Table 5).

**Table 5:** Extracellular vesicles (EVs) quantification

Sample	EVs (particles/ml)
Control	7.01 × 10⁸ (± 1.89 × 10⁸)
Long COVID	6.86 × 10⁸ (± 1.70 × 10⁸)

No statistically significant difference was observed between the groups (*p* = 0.58) (Ten independent experiments).

We then analyzed the exosome surface markers using flow cytometry. As shown in Fig. 1L-M, while the levels of most markers were comparable, we observed significant differences for specific markers. Specifically, we identified a significant overexpression of markers associated with platelet hyperactivation in EVs derived from long COVID (LC) subjects (CD29, CD41b, CD42a, and CD69).

### LC-Derived EVs drive stress response in lung cells

**Fig. 2:**
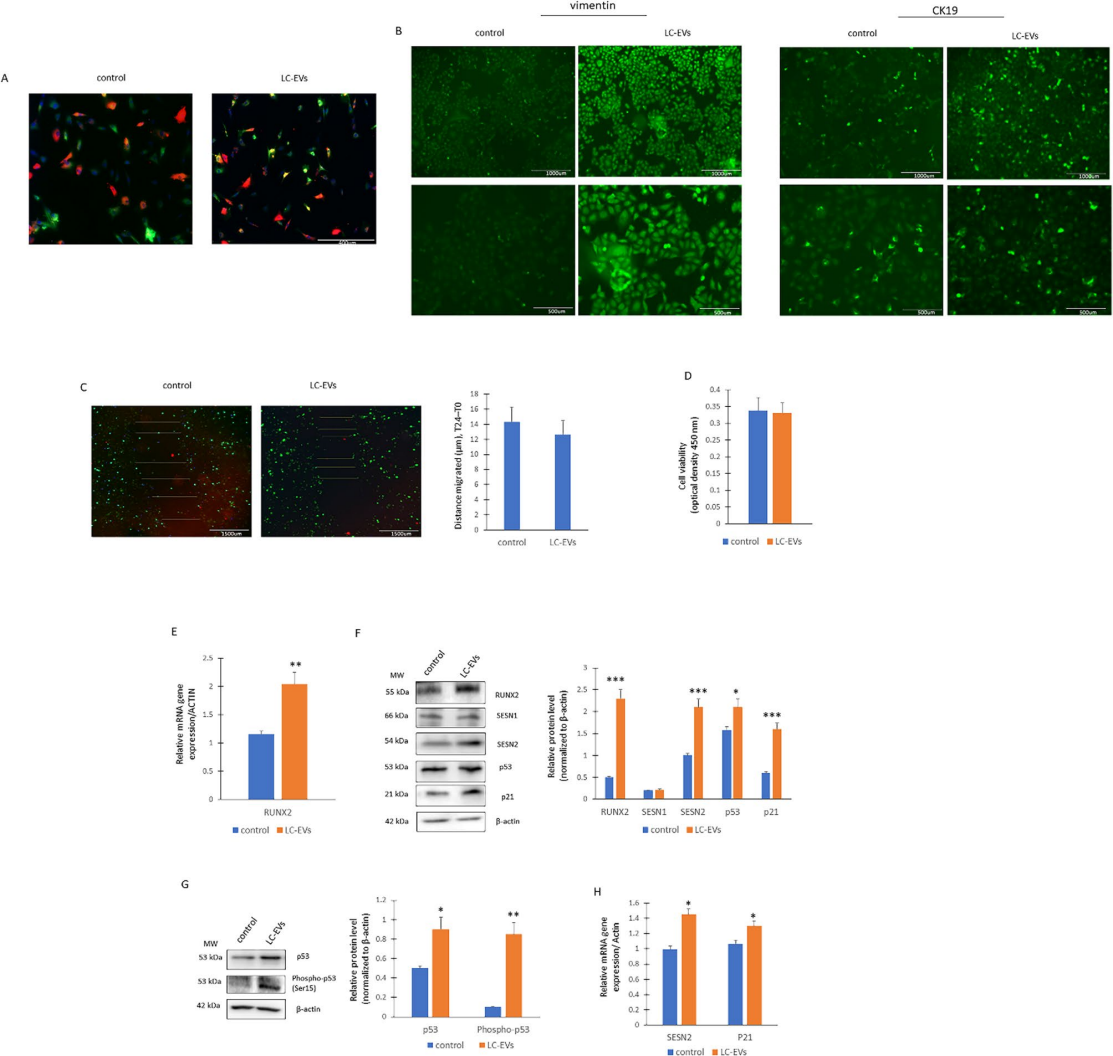
LC-EVs effects on A549 lung cells. **A** The EVs (red) from both the controls and LC crossed the cell (green) membranes and they were completely internalized by the cells after 18 h (*n* = 6 independent experiments). **B** Immunofluorescence staining for vimentin and CK19 (*n* = 3 independent experiments). **C** Scratch test to assay migration ability in EVs treated lung cells (*n* = 4 independent experiments). **D** viability cells in EVs treated lung cells (*n* = 3 independent experiments). **E** Real-Time PCR for RUNX2 in EVs treated lung cells (*n* = 3 independent experiments; **p = 0.0046). **F** WB for protein levels (on the left) and optical density of the blots (on the right); (*n* = 4 independent experiments; RUNX2, ***p = 0.0006; SESN2, ***p = 0.0008; p53, *p = 0.038; p21, ***p = 0.0009). **G** WB for protein levels (on the left) and optical density of the blots (on the right); (*n* = 3 independent experiments; p53, *p = 0.032; phosphor-p53, **p = 0.0024). **H** Real-Time PCR for SESN2 and P21 gene expression (*n* = 3 independent experiments, SESN2, *p = 0.038; p21, *p = 0.044)

**Fig. 3:**
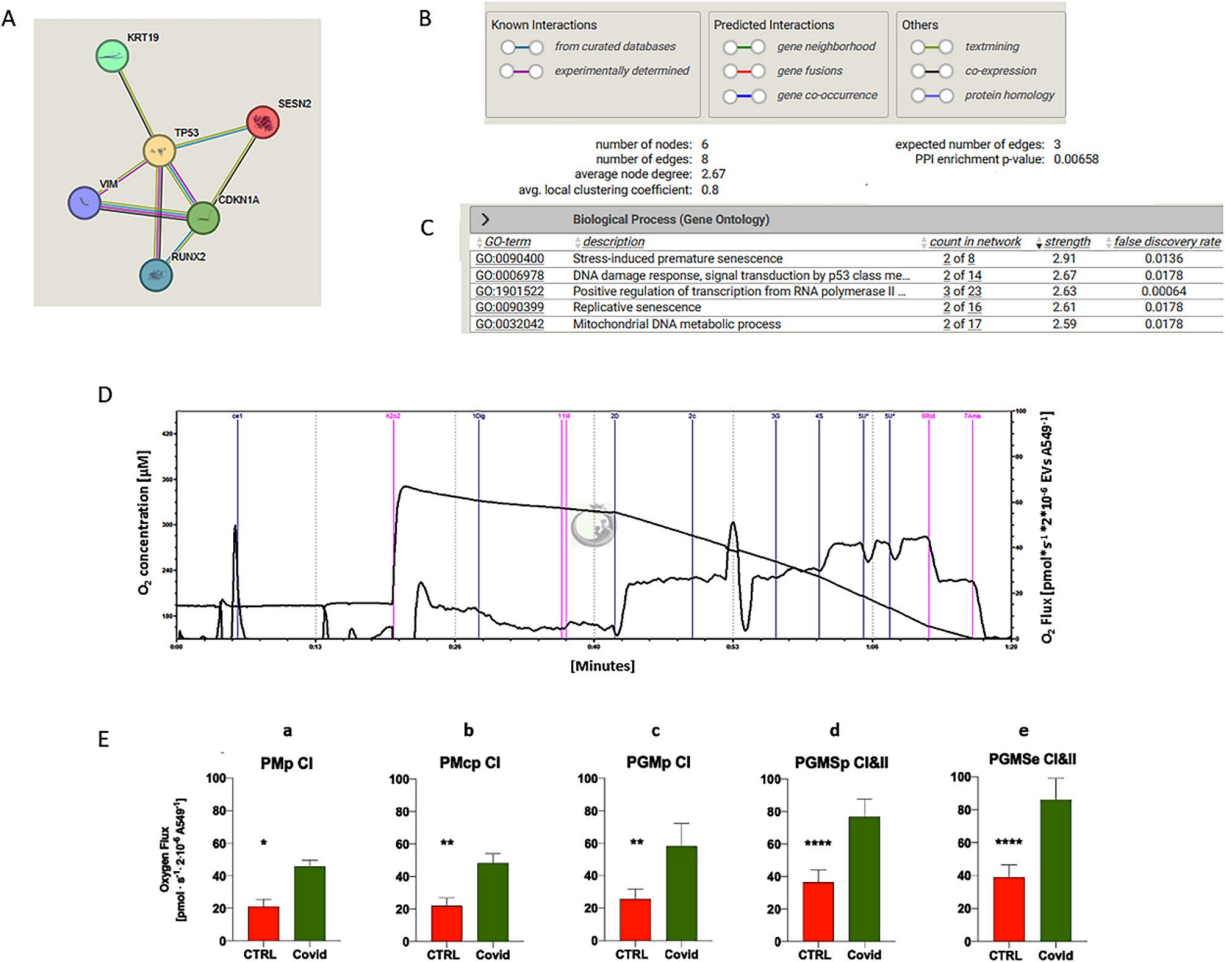
Protein interactions and respiratory capacities. Functional protein interactions identified by STRING analyses (**A**) and enrichment analysis (PPI enrichment *p*-value: 0.00658) (**B**). Biological Process identified by string analysis (**C**). Representative O2k trace (**D**) (*n = 2 independent analyses and tree replicates for each experiment*). CI uses saturating concentrations of pyruvate, malate, and glutamate as substrates (**a**, **b**, **c**). Max ETS is achieved using saturating concentrations of pyruvate, malate, ADP, glutamate, succinate (for complex II), and then titrated with CCCP to uncouple respiration to achieve maximal respiration. Complex I (**d**) and MaxETS (**e**) respiration for control and LongCovid EVs-treated A549 cells. P, pyruvate; M, malate, p, OXPHOS state, c, cytochrome c; G, glutamate; S, succinate; e, electron-transfer state; CI, complex I; CII, complex II. *= *p* < 0.05; **=*p* < 0.01; ****=*p* < 0.0001

To investigate the effects of LC-EVs on lung cells, we exposed A549 cells to EVs isolated from either control subjects or LC patients for 18 h. As shown in Fig. 3A, EVs from healthy donors and Long Covid (LC) subjects could cross cellular membranes of lung cells and disperse within the cells in an identical manner (Fig. 2A).

Notably, vimentin and CK19 were significantly overexpressed in lung cells treated with LC-EVs when compared to control EVs (Fig. 2B). However, the migratory capacity of the cells, evaluated by scratch assay (Fig. 2C) as well as cell viability (Fig. 2D) of cells treated with control or LC-EVs revealed no significant differences. Moreover, our results showed a marked overexpression of RUNX2 in cells treated with LC-derived EVs compared to controls (Fig. 2D-E). Interestingly, also SESN2 levels were increased in cells treated with LC-EVs (Fig. 2E) while SESN1 levels were similar (Fig. 2E). Protein levels of p53 and p21 were higher in cells treated with LC-EVs than in controls too (Fig. 2E). The increased p53 phosphorylation (Fig. 2G), together with the upregulation of SESN2 and p21 (Fig. 2H), supports the activation of p53 and its functional transcriptional activity.

### LC-Derived EVs disrupt mitochondrial function and cellular adaptability under metabolic stress

To explore whether the proteins modulated by LC-EVs are functionally connected, we performed an exploratory protein-protein interaction (PPI) analysis using the STRING database (https://string-db.org/; accessed on 10 September 2024). It is important to note that the list of proteins analyzed was pre-selected based on experimental modulation; therefore, this analysis is a descriptive support to identify potential shared pathways or biological functions. As shown in Fig. 3A-B, the predicted interactions within the functional network were statistically significant (p-value: 0.00658), indicating a strong functional relationship between the proteins involved. In addition, the functional enrichment analysis (protein-protein interaction enrichment) revealed the involvement of the modulated proteins in several biological processes such as stress induced senescence and mitochondrial DNA metabolic process (Fig. 3C).

**Fig. 4:**
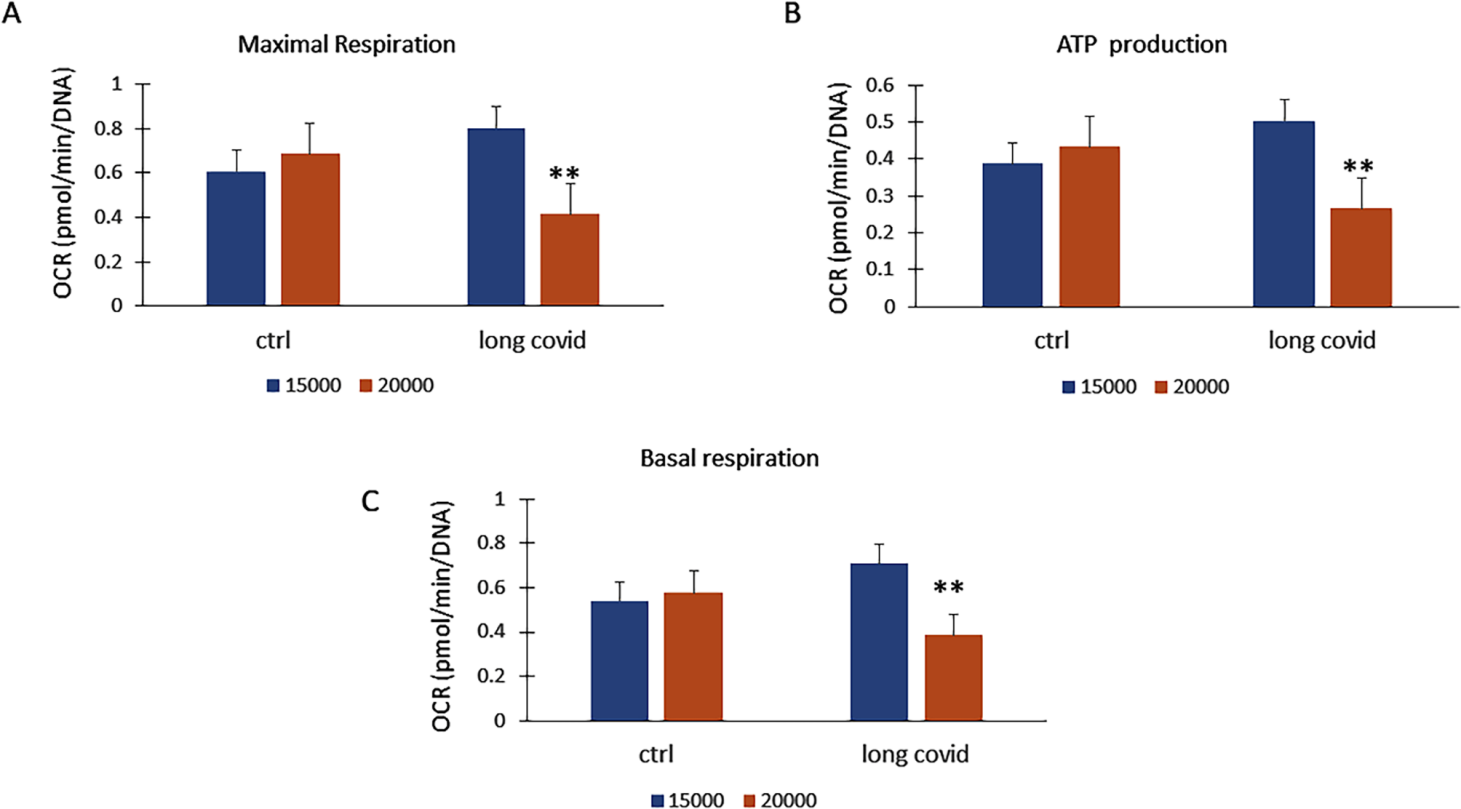
Metabolic stress response in cells treated with EVs. **A** Maximal respiration measured using the Seahorse MitoStress test decreases significantly in LC-EV-treated cells as cell density increases, whereas control vesicle-treated cells maintain stable respiration (**p = 0.0043). **B** ATP production is reduced in LC-EV-treated cells at higher cell densities, indicating impaired energy generation (**p = 0.0045). **C** Basal respiration also declines in LC-EV-treated cells under resource-limited conditions (**p = 0.0042). OCR: Oxidative consumption rate. *n* = 3 independent analyses and tree replicates for each experiment

To investigate mitochondrial function, we used high-resolution respirometry (Oroboros O2k) under hyperoxic conditions to maximize cellular respiratory capacity (Fig. 3D). After an 18-hour stimulation with EVs from control and long COVID (LC) subjects, oxygen consumption was significantly higher in LC-EV-treated cells Fig. 3E. To understand the mechanisms underlying this enhanced oxygen consumption, we conducted a MitoStress test in A549 cells using the Seahorse system. Under stress conditions caused by higher cell density—where cells compete for limited resources such as oxygen and nutrients—the metabolic capacity of LC-EV-treated cells declined. Specifically, as cell density increased, Maximal Respiration significantly decreased in these cells, whereas cells treated with control vesicles maintained stable Maximal Respiration Fig. 4A. This suggests that LC-EVs impair the cells’ ability to adapt to resource-limited conditions. Accordingly, ATP production (Fig. 4B) and basal respiration (Fig. 4C) were also reduced at higher cell densities in the presence of LC-EVs, further supporting the idea that these vesicles negatively impact mitochondrial adaptability in response to metabolic stress.

Expanding the number of nodes in the analysis of protein interactions has allowed us to identify additional processes, including apoptotic cell death triggered by DNA damage (Figure S7).

### RUNX2 overexpression and hsa-miR-204-5p modulation drive p53/p21 pathway and SESN2 activation in A549 lung cells

**Fig. 5:**
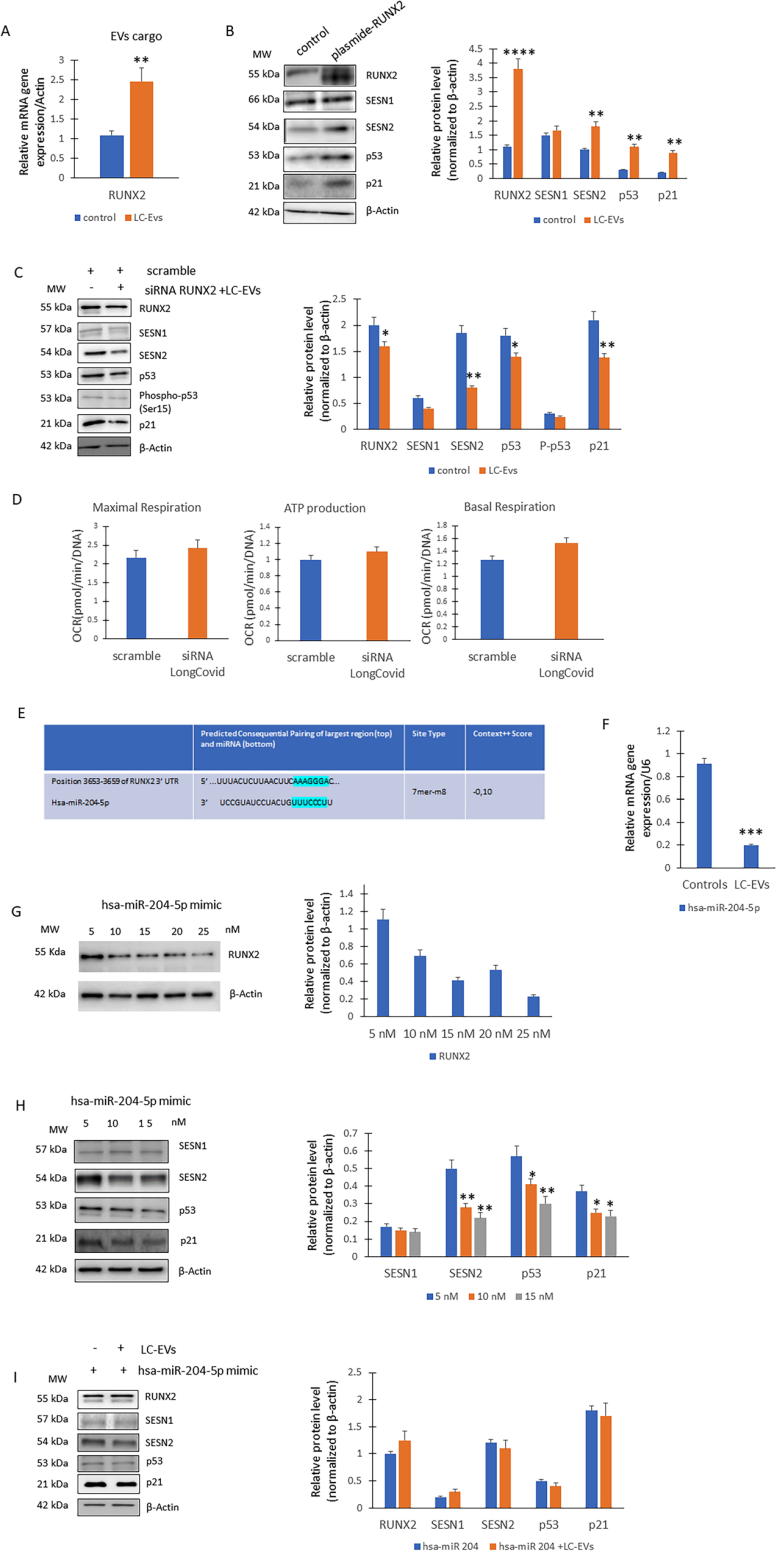
Hsa-miR-204-5p/RUNX2. Increased RUNX2 gene expression in cargo LC EVs compared to controls (*n* = 3 independent experiments; **p = 0.0046 vs. control) (**A**). WB for proteins levels (on the left) and optical density of the blots (on the right) in control or forced RUNX2 expression on A549 cells (*n* = 3 independent experiments; RUNX2, ****p = 0.00008; SESN2, **p = 0.0048; p53, **p = 0.0038; p21, **p = 0.0036 vs. controls) (**B**). WB for proteins levels (on the left) and optical density of the blots (on the right) in scramble (control) and RUNX2 silenced cells in presence of LC-EVs (*n* = 3 independent experiments; RUNX2, *p = 0.045; SESN2, **p = 0.0031; p53, *p = 0.042; p21, **p = 0.042) (**C**). Metabolic stress response in scramble (control) and RUNX2 silenced cells in presence of LC-EVs (**D**). Putative hsa-miR-204-5p binding region in RUNX2 (Prediction score in Target Scan database: 0.10) (**E**). Real-Time PCR for cargo miR 204 in controls and LC subject (*n* = 3 independent experiments; ***p = 0.0008) (**F**). WB (on the left) and optical density of the blots (on the right) for RUNX2 protein levels on A549 cells transfected with mimic hsa-miR-204-5p at concentrations ranging from 5 to 25 nM (*n* = 3 independent experiments) (**G**). WB for proteins levels (on the left) and optical density of the blots (on the right) in cells treated with hsa-miR-204-5p (*n* = 3 independent experiments; SESN2 10 nM, **p = 0.0042; SESN2 15nM, **p = 0.0038; vs. SESN2 5nM. P53 10 nM, *p = 0.047; p53 15 nM, **p = 0.0037; vs. p53 5nM. P21 10nM, *p = 0.048. p21 15 nM, *p = 0.048, vs. p21 5 nM (**H**). WB for proteins levels (on the left) and optical density of the blots (on the right) in cells treated with hsa-miR-204-5p without or with LC-EVs (*n* = 3 independent experiments) (**I**)

To understand the role of LC-EVs in affecting A549 lung cells, we analyzed the RUNX2 mRNA levels in the cargo of LC-EVs and controls. Thus, we observed increased RUNX2 gene expression in cargo LC EVs compared to controls (Fig. 5A). Therefore, to investigate the role of RUNX2 in modulating the increase in SESN2 and the activation of the p53/p21 pathway, we overexpressed RUNX2 in A549 cells. Forced expression of RUNX2 induced the activation of the p53/p21 pathway and resulted in increased SESN2 levels (Fig. 5B), as observed in lung cells treated with LC-EVs. In addition, to determine whether the effect observed after LC-EV treatment was mediated by RUNX2, we exposed RUNX2-silenced A549 cells to LC-EVs and subsequently analyzed the cellular markers as well metabolic stress response. As shown in the Fig. 5C, RUNX2 downregulation leads to a reduction in stress protein levels even in the presence of LC-EVs, when compared with scramble/control cells. With respect to metabolic stress, RUNX2 downregulation was able to restore EV-treated cells to scramble/control levels (Fig. 5D). Thus, we explored the role of molecules involved in the post-transcriptional regulation of RUNX2. Through an in silico analysis using TargetScan, we identified a putative hsa-miR-204-5p binding site in the 3′-UTR of RUNX2 mRNA. In particular, the binding site is in an evolutionarily conserved region (Fig. 5E). Thus, by analyzing the hsa-miR-204-5p content in EVs cargo, we observed reduced expression of hsa-miR-204-5p in LC-EVs compared to controls (Fig. 5F**)**. Therefore, to test the role of hsa-miR-204-5p in modulating RUNX2 expression, hsa-miR-204-5p (mimic hsa-miR-204-5p) was delivered into A545 cells via Lipofectamine-mediated transfection at concentrations ranging from 5 to 25 nM. As shown in Fig. 5G, RUNX2 protein levels decreased in response to increasing concentrations of mimic hsa-miR-204-5p. Similarly, higher concentrations of mimic hsa-miR-204-5p (ranging from 5 to 15 nM) also reduced the protein levels of SESN2, P53, and p21. (Fig. 5H). However, in the presence of high levels of miR-204, the addition of LC-EVs induced only a slight increase in RUNX2, while the other stress markers remained unchanged (Fig. 5I).

### LC-EVs elicit Stress-Induced signaling in IMR90 lung fibroblasts

**Fig. 6:**
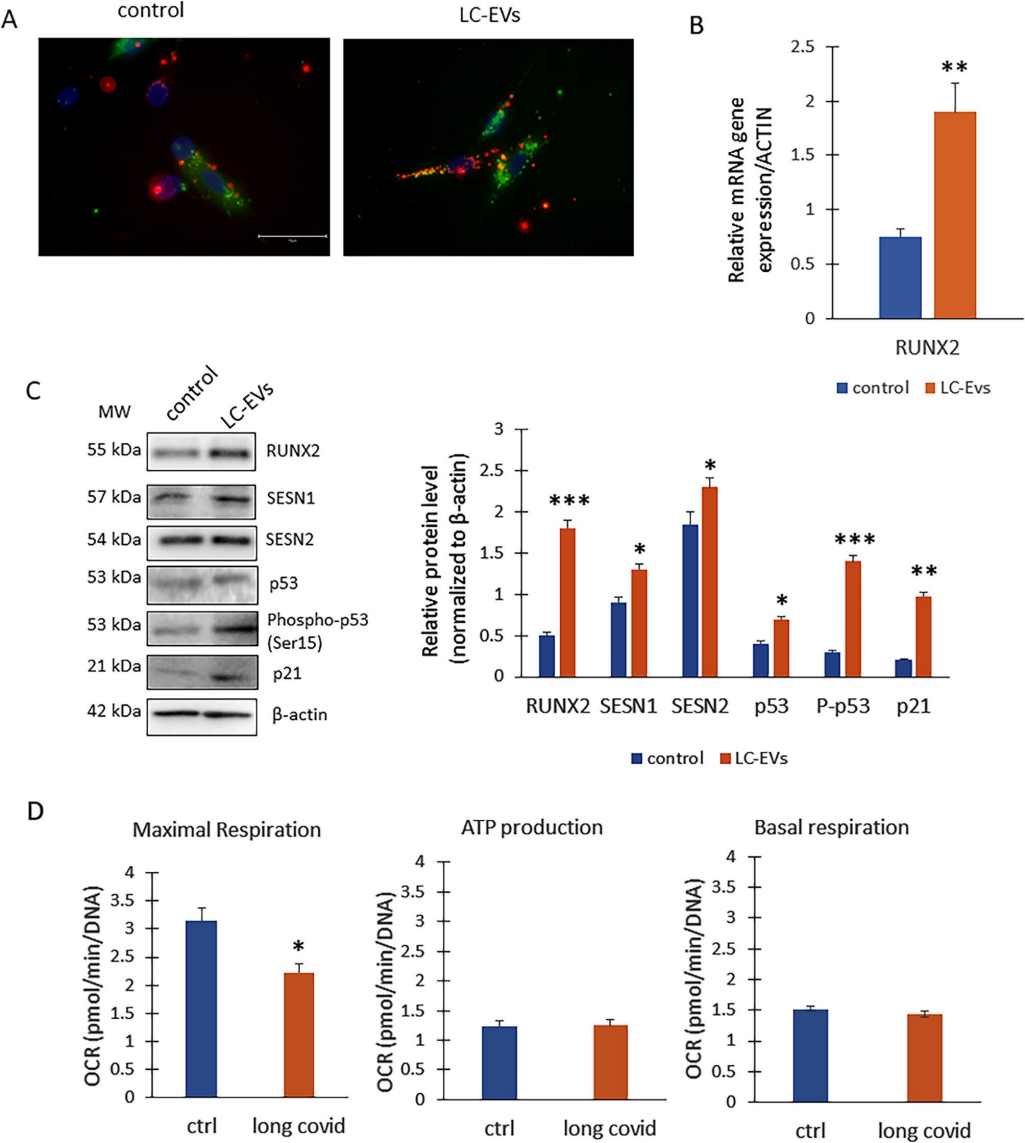
Effects of EVs on IMR90 fibroblast cells. **A** The EVs (red) from both the controls and LC crossed the cell (green) membranes and were completely internalized into the IMR90 cells (*n* = 3 independent experiments). **B** Real-Time PCR for RUNX2 (**p = 0.004 vs. control) and **C** WB for protein levels (on the left) and optical density of the blots (on the right; RUNX2, ***p = 0.0008; SESN1*p = 0.034 SESN2*p = 0.036; p53, *p = 0.041; Pp53, ***p = 0.00084; p21, **p = 0.048 vs. controls) of EVs treated IMR90 (*n* = 3 independent experiments for both Real time and WB). **D** Metabolic stress response in cells treated with EVs (*n* = 3 independent experiments, *p = 0.046)

In IMR90 fibroblast lung cells, exposure to LC-EVs (Fig. 6A), impaired cellular homeostasis, as evidenced by a pronounced increase in RUNX2 expression (Fig. 6B) together with the upregulation of stress-associated markers (Fig. 6C). At the metabolic level, LC-EVs detrimentally affected mitochondrial function by reducing maximal respiratory capacity (Fig. 6D), while ATP levels and basal respiration remained unchanged, suggesting a selective impairment of bioenergetic flexibility without compromising basal energy supply.

### RUNX2 Overexpression and Stress Response Activation in Endothelial and Aortic Smooth Muscle Cells Exposed to LC-Derived EVs

**Fig. 7:**
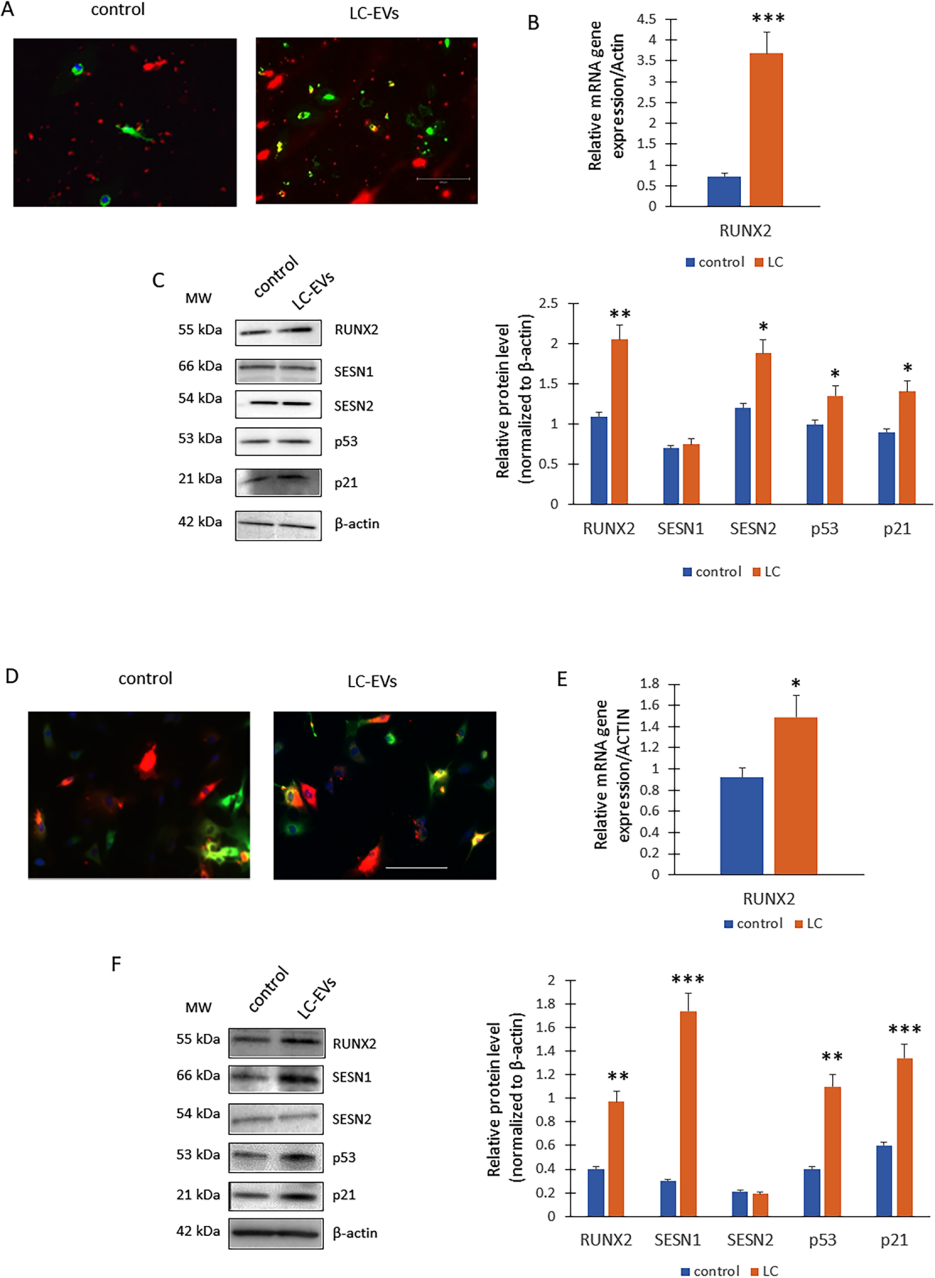
Effects of EVs on endothelial cells (EC) and ASMCs. **A** The EVs (red) from both the controls and LC crossed the cell (green) membranes and were completely internalized into the EC cells (*n* = 3 independent experiments). **B** Real-Time PCR for RUNX2 (***p = 0.0007 vs. control) and (**C**) WB for protein levels (on the left) and optical density of the blots (on the right; RUNX2, **p = 0.0038; SESN2*p = 0.021; p53, *p = 0.043; p21, *p = 0.041 vs. controls) of EVs treated ECs (*n* = 3 independent experiments for both Real time and WB). (**D**) The EVs (red) from both the controls and LC crossed the cell (green) membranes and were completely internalized into the ASMCs (*n* = 3 independent experiments). **E** Real-Time PCR for RUNX2 (*p = 0.047) and (**F**) WB for protein levels (on the left) and optical density of the blots (on the right, RUNX2, **p = 0.0035; SESN1, ***p = 0.0002; p53, **p = 0.0027; p21, ***p = 0.00048 vs. controls) of EVs treated ASMCs (*n* = 3 independent experiments for both Real time and WB)

We also observed a significant increase in RUNX2 expression in both endothelial cells (EC) and aortic smooth muscle cells (ASMC). As shown in Fig. 7A, EVs from both control and long COVID patients were able to enter endothelial cells, and after 18 h, they were fully localized within the cytoplasm.

Both gene expression (Fig. 7B) and RUNX2 protein levels (Fig. 7C) were higher in cells treated with LC-EVs compared to controls. Additionally, SESN1 levels were similar, while p53, p21, and SESN2 levels were higher in endothelial cells exposed to LC-EVs compared to controls. Similarly, EVs from both control and long COVID subjects efficiently entered ASMCs, with complete internalization observed within 18 h (Fig. 7D). In ASMC we also observed an upregulation of the RUNX2 gene (Fig. 7E) and related protein levels (Fig. 7F) following treatment with LC-EVs. However, we observed higher levels of SESN1 in ASMC treated with LC-EVs compared to controls, while SESN2 levels were similar in both groups **(**Fig. 7F**)**. As observed in lung cells and endothelial cells, we observed higher levels of p53 and p21 in cells treated with LC-EVscompared to controls **(**Fig. 7F**)**.

### RUNX2 and stress pathway activation in mesenchymal stem cells exposed to long COVID-Derived EVs

**Fig. 8:**
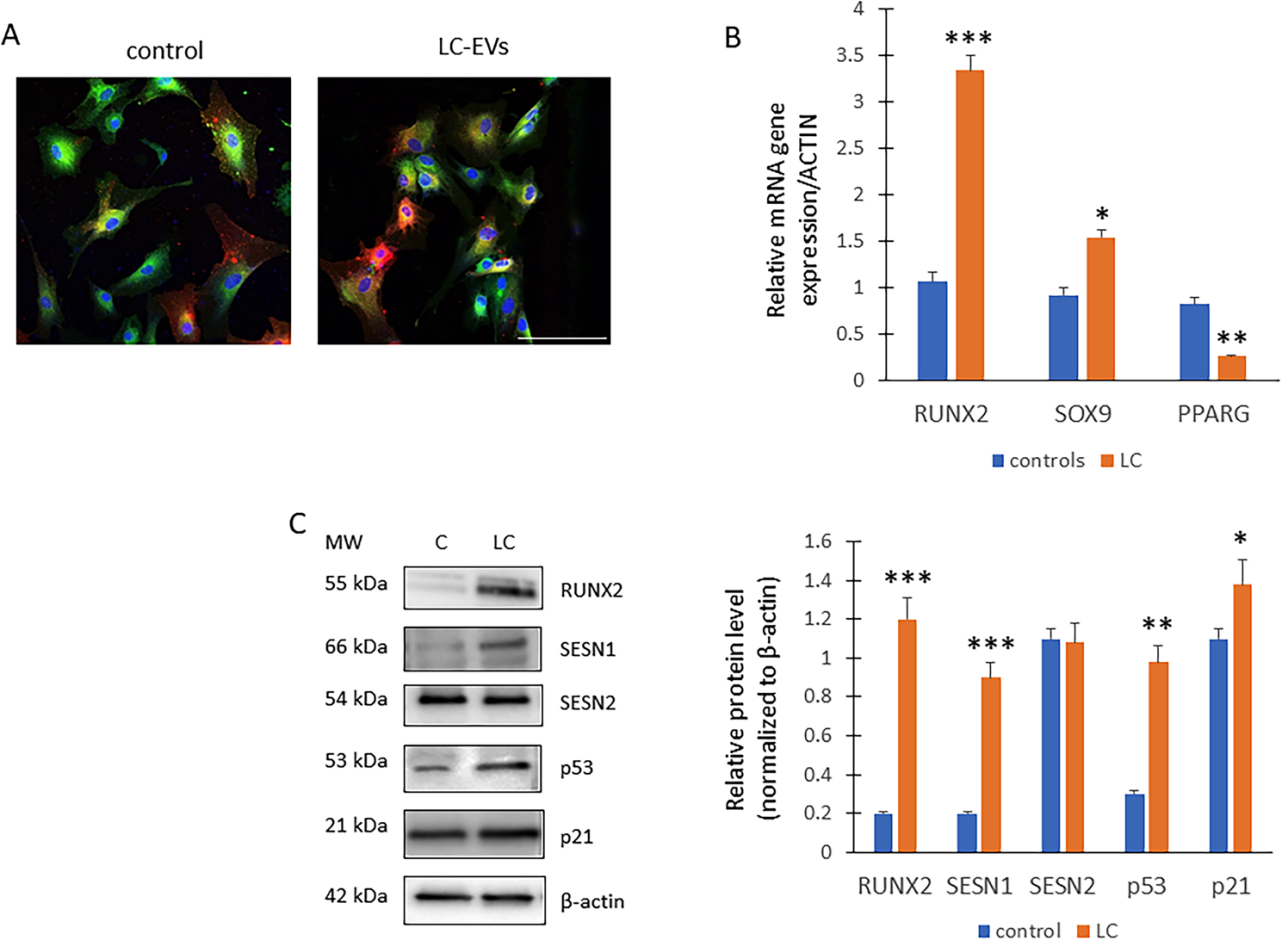
Effects of LC-EVs on hMSCs. **A** The EVs (red) from both the controls and LC crossed the cell (green) membranes and were completely internalized by the cells after 18 h (*n* = 6 independent experiments). **B** Real-Time PCR for RUNX2 (***p = 0.0002 vs. control), SOX9 (*p = 0.047 vs. control) and PPARG2 (**p = 0.0038 vs. control). **C** WB for protein analyses (on the left) and optical density of the blots (on the right; RUNX2, ***p = 0.0007; SESN1, ***p = 0.0009; p53, **p = 0.0035; p21, *p = 0.048) (*n* = 3 independent experiments for both Real time and WB)

We also investigated the impact of LC-EV on a human MSCs line. Our data demonstrated that similarly to the above-investigated cells, EVs from both control and long COVID sources entered the mesenchymal stem cells, with complete internalization observed within 18 h (Fig. 8A).

In hMSCs we also observed increased gene expression levels of RUNX2 following treatment with long COVID-derived EVs compared to controls (Fig. 8B). Additionally, we observed an increase in SOX9 gene expression and a decrease in PPARG expression (Fig. 8B). Additionally, we observed higher levels of RUNX2, SESN1, p53 and p21 proteins in MSCs treated with LC-derived EVs (Fig. 8C**).**

## Discussion

Our data strongly suggest a key pathogenic mechanism of long COVID mediated by vesicles (EVs). Using cytofluorimetric analysis, we identified a significant overexpression of markers associated with platelet hyperactivation in LC-EVs. Specifically, we observed elevated levels of CD29, CD41b, CD42a, and CD69, which have been reported in the literature as key players in platelet aggregation [43, 44]. Notably, the downregulation of these markers is implicated in hemorrhagic shock, emphasizing their importance in hemostasis [45].Furthermore, it has been reported that LC individuals display signs of altered platelet activity [46]. It is well-established that vascular endothelial cell damage leads to an increased expression of adhesion molecules that interact with platelets, including von Willebrand factor (VWF), selectins, and integrins, while concurrently reducing the expression of anti-thrombogenic factors. This endothelial dysfunction facilitates platelet activation and thrombogenesis. In line with these observations, persistently elevated levels of von Willebrand factor (VWF) antigen and its pro-peptide in individuals recovering from COVID-19 were identified, lasting up to six weeks after symptom resolution [47]. Likewise, Meijenfeldt et al. reported heightened concentrations of plasminogen activator inhibitor type 1 in the serum of COVID-19 patients even four months post-infection. Thus, these studies highlight the extended pro-thrombotic condition observed in individuals experiencing prolonged symptoms following SARS-CoV-2 infection [48, 49].

By exposing lung cells to EVs from both healthy donors and LC patients, we observed that these vesicles effectively crossed the cellular membrane and dispersed within the cell’s cytoplasm. This observation is consistent across both control and LC-EVs, suggesting a fundamental ability of these vesicles to enter cells regardless of their origin. As vimentin is an indicator of fibroblastic activation and cellular migration, while CK19 is a marker of cellular differentiation associated with neoplastic and regenerative processes ref [50], we analyzed their expression in EVs treated lung cells. Thus, both vimentin and CK19 increased in LC-EVs treated cells. Notably, vimentin and CK19, are associated with epithelial-mesenchymal transition (EMT) and tumor invasion ref [51]. However, cell migration assays did not reveal an increase in migratory capacity, suggesting that the upregulation of vimentin and CK19 may instead reflect enhanced fibrotic potential or be related to an adaptive response to stress, as previously reported ref [52, 53, 54]. In addition, RUNX2 has been reported to be linked to profibrotic signaling contributing to pulmonary fibrosis [19, 54] and invasion as well as in inflammation-oriented lung diseases [55]. Thus, the increased RUNX2 expression in LC-EVs-treated cells further highlights the role of RUNX2 in profibrotic signalling and pulmonary fibrosis [19, 56]. The association of RUNX2 with fibrotic features in lung cells strengthens its importance as a target in understanding the lung pathology observed in LC patients.

We therefore assessed the levels of sestrins, proteins activated under stress conditions and involved in emphysema processes and expressed in cells from Chronic obstructive pulmonary disease (COPD) patients [57]. Sestrins are a family of highly conserved proteins that play a crucial role in cellular responses to negative stimuli, including oxidative stress, nutrient deprivation, and other forms of cellular stress [58]. They function primarily as stress sensors and regulators of cellular homeostasis. By modulating various signaling pathways, including the mammalian target of rapamycin (mTOR) and AMP-activated protein kinase (AMPK), sestrins help maintain energy balance and promote cell survival under adverse conditions [59]. Their dysregulation has been implicated in several diseases, including cancer and metabolic disorders, highlighting their potential as therapeutic targets in stress-related pathologies [59]. In lung cells, we observed an increase in SESN2 levels in response to LC-EVs, suggesting a cellular response to adverse conditions. Indeed, SESN2 plays a protective role against various stress stimuli, such as genotoxic, energetic, and oxidative stress [60]. Since Sestrins are transcriptionally activated by p53 [11], to evaluate p53 activation, we analyzed phosphorylated p53 (Phospho-p53) and its canonical target p21 in cells treated with LC-EVs. Thus, we observed that both p53 and its phosphorylated form, as well as p21, were increased in cells exposed to LC-EVs. These findings indicate activation of the p53 pathway, which is commonly involved in cellular responses to DNA damage, oxidative stress, and other forms of cellular stress [61]. Upregulation of p21, a downstream target of p53, typically results in cell cycle arrest, allowing the cell time to repair damage or, if unsuccessful, to undergo senescence or apoptosis [61]. In addition, the involvement of Sestrins in this process may suggest their role in managing oxidative stress and regulating pathways associated with cellular survival and metabolism.

Our bioinformatic analysis of protein-protein interactions further supports these findings, with significant functional association between the altered proteins, particularly highlighting mitochondrial DNA metabolic processes. As mitochondrial structural abnormalities have been reported in Long COVID patients [40], we focused on investigating the mitochondrial DNA metabolic process among the identified biological processes (GO:0032042).

Notably, we observed that LC-EVs have a dual effect on mitochondrial function. LC-EVs-treated cells analyzed under oxygen-favorable conditions exhibited higher oxygen consumption, as measured by the Oroboros O2k oxygraph. This indicates an increase in mitochondrial respiration, which could be due to enhanced metabolic activity or a compensatory response to stress induced by LC-EVs. However, when metabolic stress is exacerbated by higher cell density, the ability of LC-EV-treated cells to sustain maximal respiration significantly declines (Seahorse MitoStress test). This suggests that while LC-EVs may initially stimulate mitochondrial activity, they ultimately impair the cells’ adaptive metabolic flexibility, making them more vulnerable under conditions where resources such as oxygen and nutrients are limited. A key consequence of this impaired adaptability is the reduction in ATP production and basal respiration at higher cell densities. Since maximal respiration reflects the full capacity of the electron transport chain, its decline in LC-EVs-treated cells implies that mitochondria become less efficient in generating ATP under stress. In contrast, cells treated with control vesicles maintain stable respiration and ATP production, indicating that their mitochondrial function remains more resilient. Overall, these findings suggest that LC-EVs disrupt the metabolic homeostasis of recipient cells, particularly in conditions of high metabolic demand. The effects of LC-EVs on mitochondrial respiration may reflect a context-dependent cellular response. In hyperoxic environments, LC-EVs might promote mitochondrial activity as part of a compensatory mechanism aimed at sustaining energy production and cellular homeostasis. Conversely, under stress conditions the LC-EVs-mediated signaling could contribute to mitochondrial dysfunction, acting as a maladaptive or pathological response. This duality suggests that EVs may play a pivotal role in the balance between adaptation and degeneration, particularly in the context of Long COVID, where chronic dysregulation of cellular stress responses is likely involved [62]. In addition, the observation that LC-EVs provoke cellular stress also in IMR90 fibroblasts highlights their potential detrimental role in modulating cell homeostasis. From a metabolic perspective, the reduction in maximal respiratory capacity indicates an impairment in mitochondrial flexibility and resilience under stress conditions. Interestingly, basal respiration and ATP levels remained unaffected, implying that while LC-EVs compromise the ability of cells to meet increased energetic demands, they do not disrupt the maintenance of basic energy supply. This selective impairment may predispose cells to vulnerability under additional stressors, thus amplifying the deleterious consequences of LC-EV exposure. This could have important implications for understanding how long COVID affects cellular energy metabolism, potentially contributing to fatigue and other persistent symptoms observed in patients. These findings can also be detected when long COVID patients report fatigue and exercise-induced leg discomfort [63]. In fact, We described that this symptom may be present even at low intensity exercise like the six Minute Walking Test.

To identify a potential mechanism by which LC-EVs induce cellular alterations in RUNX2/p53/sestrins pathway, we analysed the mRNA RUNX2 levels carried by EVs. Therefore, we observed an increase in RUNX2 mRNA levels in LC-EVs. Considering that various studies have reported different levels of gene expression for miRNAs present in the EV cargo [64, 65], we investigated the levels of microRNA-204. This microRNA was selected based on an in silico analysis using TargetScan, as well as previous evidence showing its role in modulating RUNX2 [66]. In particular, it has been demonstrated that hsa-miR-204-5p is a well-known direct negative regulator of RUNX2, targeting its 3’UTR in various cell types ref [67, 68]. We observed that the levels of hsa-miR-204-5p were lower in the cargo of LC-EVs, which was consistent with the increased levels of RUNX2 seen in LC-EVs and in cells treated with LC-EVs. The increase in RUNX2 levels in recipient cells was also responsible for the activation of the p53/p21/Sestrins pathway, as confirmed by inducing forced upregulation of RUNX2 in cells by transfection with a RUNX2 overexpressing plasmid. Thus, our data suggest that this RUNX2 upregulation is, at least in part, due to reduced levels of hsa-miR-204-5p in LC-EVs. In addition, hsa-miR-204 can modulate p53 indirectly in a context-dependent manner—for instance, in cardiomyocytes, hsa-miR-204 inhibits apoptosis by targeting SIRT1, thereby reducing p53 activation ref [69]. In our study, we observed that both RUNX2 and p53 levels are elevated following treatment with LC-EVs. This finding is consistent with previous studies showing that forced overexpression of RUNX2 can induce senescence-like growth arrest through a p53-dependent mechanism ref [70, 71]. Therefore, we interpret the concurrent upregulation of RUNX2 and p53 as a cellular stress response to signals delivered by LC-EVs. Moreover, by transfecting the cells with a miR-204 mimic, we observed that the upregulation of miR-204 leads to a reduction in cellular stress markers. Although miR-204 levels were high, LC-EV treatment still induced an increase in RUNX2 expression; however, this was not sufficient to drive the upregulation of stress markers. These findings suggest that miR-204 plays a protective role by limiting the activation of stress pathways. Taken together, these observations suggest that the regulatory action of miR-204 counterbalances the pro-stress influence of RUNX2 and may represent a key mechanism in maintaining cellular homeostasis.

One limitation of this study is the lack of direct evidence for the efficiency and functional relevance of hsa-miR-204-5p transfer via EVs in repressing RUNX2 in recipient cells. Given that EV-mediated miRNA delivery can vary depending on cell type and context, future studies should include the use of fluorescently labeled hsa-miR-204-5p mimics or reporter assays (e.g., luciferase constructs containing the RUNX2 3’UTR) to directly assess hsa-miR-204-5p delivery and its functional repression of RUNX2. However, it could not be excluded that other miRNA/regulatory factors may be involved in the observed signalling effect. In addition to lung cells, our study extends to endothelial, aortic smooth muscle cells (ASMC) and hMSCs, where we also observed increased RUNX2 expression after treatment with LC-EVs. However, in ASMC and mesenchymal cells, we found increased levels of SESN1 when treated with LC-EVs. These findings could be due to specific mechanisms of each cell type selectively upregulating one of the Sestrins to avoid redundant responses [58]. Since both SESN1 and SESN2 modulate mTOR signalling and antioxidant responses, cells may optimize the expression of a specific Sestrin depending on the particular stress context, allowing them to fine-tune their stress adaptation mechanisms. Thus, the upregulation of RUNX2, p53, p21, and SESN1 or 2 in these cells suggests a systemic effect of LC-EVs, affecting various cell types involved in cardiovascular and pulmonary functions.

Finally, our investigation into human mesenchymal stem cells (MSCs) revealed an increase in RUNX2 gene expression, together with changes in SOX9 and PPARG expression levels. It has also been reported that an increase in both RUNX2 and SOX9 may be associated with hypoxia-induced mineralization ref [72]. Our data suggest that LC-EVsmay induce hypoxic conditions, leading to increased levels of these transcription factors, which may contribute to abnormal tissue mineralization and fibrosis development. Notably, these results are important because mesenchymal stem cells (MSCs) have been considered as a promising potential therapy for COVID-19 ref [73, 74]. However, since LC-EVscan alter MSC function, caution is needed. Injecting MSCs into patients with abnormal EVs might not be the best strategy, as it could affect their therapeutic effects. In fact, MSCs could become fibrotic, potentially exacerbating the profibrotic environment induced by LC-EVs. This underscores the need for caution when using cell therapies in such conditions.

## Conclusions

Our study demonstrates that LC-EVs carry a distinct molecular signature capable of inducing significant cellular alterations. The LC-EVs are enriched in markers of platelet activation and contribute to a pro-thrombotic environment, aligning with vascular dysfunction observed in LC. Upon entering target cells, LC-EVs promote the expression of RUNX2 and activate stress-related pathways involving p53, p21, and Sestrins, indicating a response to cellular stress. This is further supported by functional data showing impaired mitochondrial adaptability under metabolic strain, potentially explaining fatigue and exercise intolerance in LC patients. We also identified a regulatory mechanism involving decreased hsa-miR-204-5p and increased RUNX2 mRNA within LC-EVs, suggesting a role in modulating recipient cell gene expression. Notably, these effects were observed across multiple cell types, including lung, endothelial, smooth muscle, and mesenchymal stem cells, pointing to a systemic impact.

Given that LC-EVs alter mesenchymal stem cell function, our findings call for caution in considering MSC-based therapies for long COVID, as these cells may be differentiated toward profibrotic phenotypes. In summary, LC-EVs appear to play a key pathogenic role in long COVID by disrupting cellular homeostasis and promoting chronic dysfunction in multiple tissues.

In conclusion, our study provides a detailed characterization of LC-EVs and healthy controls. Despite similar EV quantities between groups, we identified distinct molecular signatures and functional effects associated with LC-EVs, including alterations in cargo composition and their impact on recipient cell responses. These findings suggest that qualitative changes in EV content—rather than quantity alone—may contribute to the persistence of post-viral symptoms. Our results advance current understanding of EV-mediated mechanisms in post-viral syndromes and support the potential use of EV profiling as a biomarker strategy. A limitation of this study is that EV-associated molecular analyses were performed on pooled samples rather than on individual patient samples. As a result, we were not able to perform correlation analyses with clinical indices such as 6MWT performance or DLCO. Future studies with subject-level EV characterization will be required to establish these clinically relevant associations and to assess the effects of EV-LCs in larger patient cohorts. Moreover, we did not evaluate primary alveolar epithelial cells, and pulmonary artery smooth muscle cells were not included. Incorporating these primary pulmonary cell types in future studies would provide additional physiological insights into the effects of extracellular vesicles in Long COVID.

## Supplementary Information

Supplementary Material 1.

Supplementary Material 2.

Supplementary Material 3.

Supplementary Material 4.

## Data Availability

Materials related to this study can be obtained from the corresponding author with a reasonable request.
