# Supplementary material for: Extracellular vesicles from long COVID patients promote RUNX2-mediated cellular stress via dysregulated miR-204 and p53 pathway activation
Source: Cell Commun Signal. 2025 Nov 26;23:508. doi: 10.1186/s12964-025-02502-7 (PMC12659154; doi:10.1186/s12964-025-02502-7)
Supplement: Supplementary file 1 — Supplementary Material 1. [file 12964_2025_2502_MOESM1_ESM.pdf]

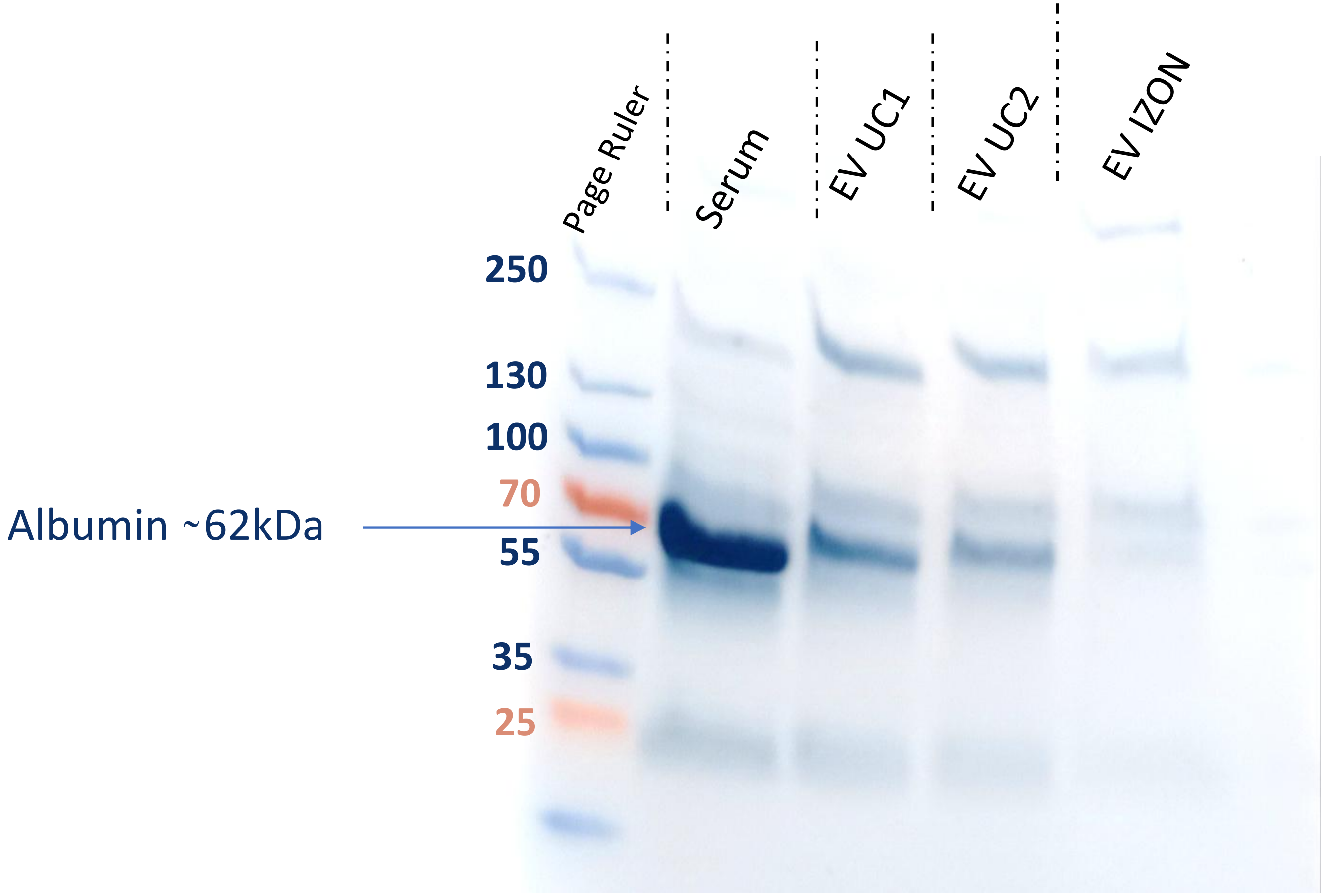

Figure S1. Detection of proteins evaluated by amido black in serum (lane a), differential ultracentrifugation lanes (b and c) and by Izon columns (d)
Serum = raw serum diluted 1:100

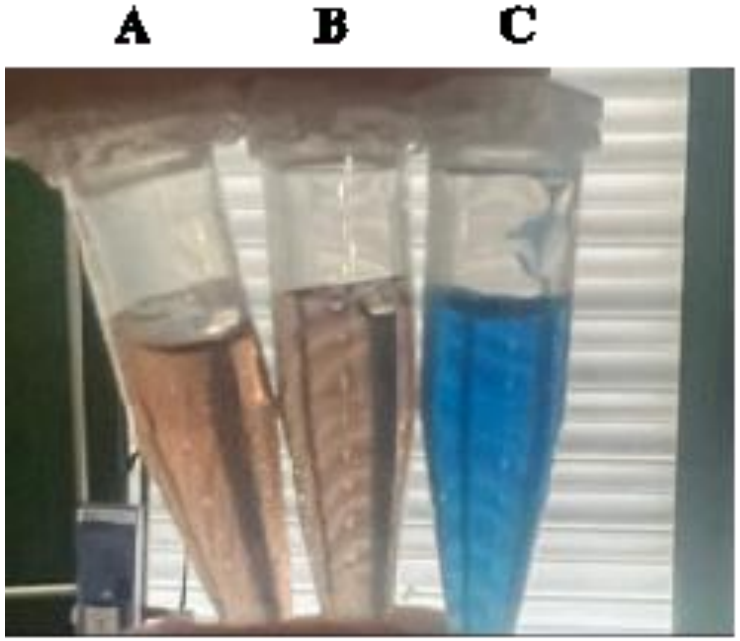

Figure S2. Assessment of protein contamination in extracellular vesicle (EV) fractions: (A) Bradford colorimetric assay negative control, (B) EV-containing fraction (0–2 mL) showing no color change, indicating absence of detectable serum proteins. (C) Serum protein-containing fraction (3.2–8 mL) showing strong blue coloration, confirming the presence of protein contaminants.

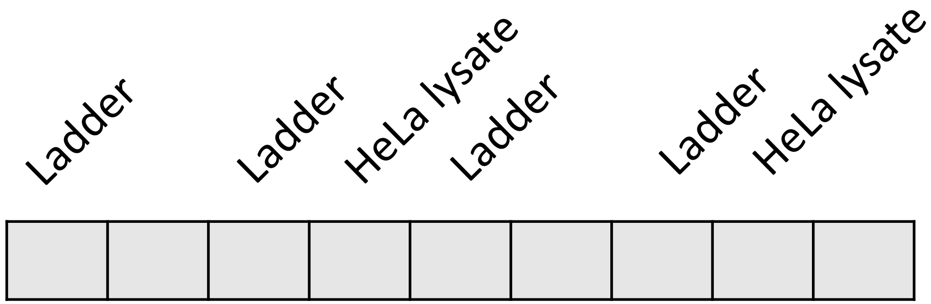

	Starting serum volume (µl)	Protein concentration after isolation (µg/µl)	Total Protein (µg)
Serum-EVs 107	456	1.279	51.14733352
Serum-EVs 110	370	1.212	48.46617088
Serum-EVs 113	480	1.553	62.11763911

40µg of protein loaded

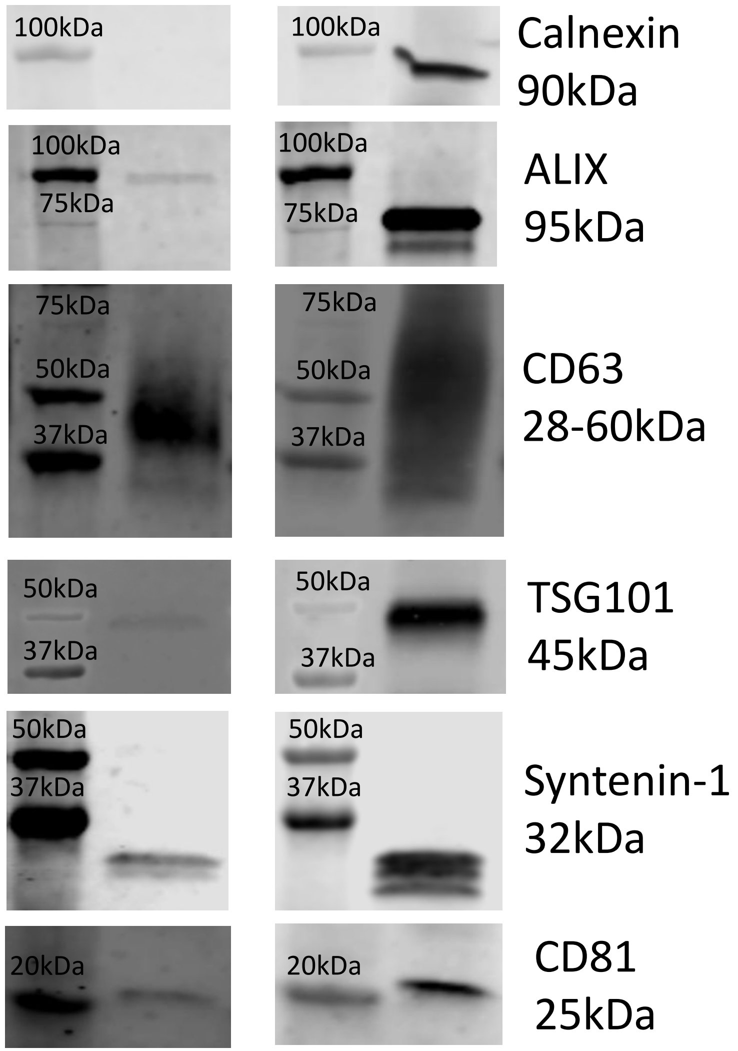

Figure S3: Standard Evs markers

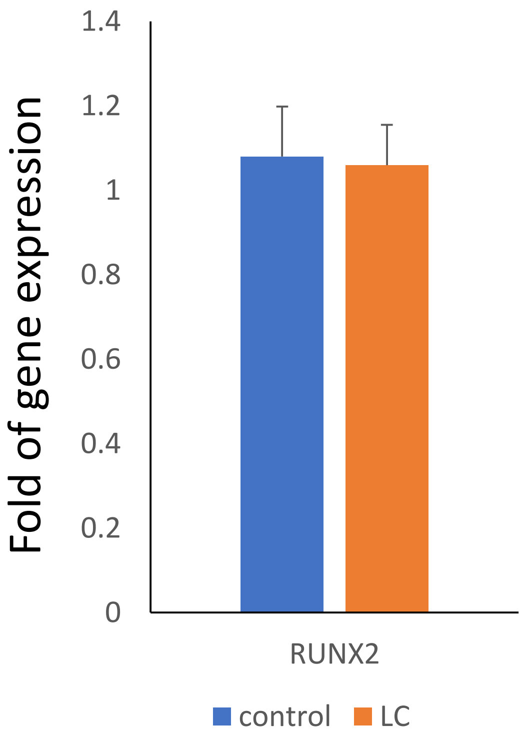

Figure S4: Treatment of A549 cells with the later fractions ("protein-rich fractions") of EVs isolation of control or Long COVID samples did not affect RUNX2 expression.

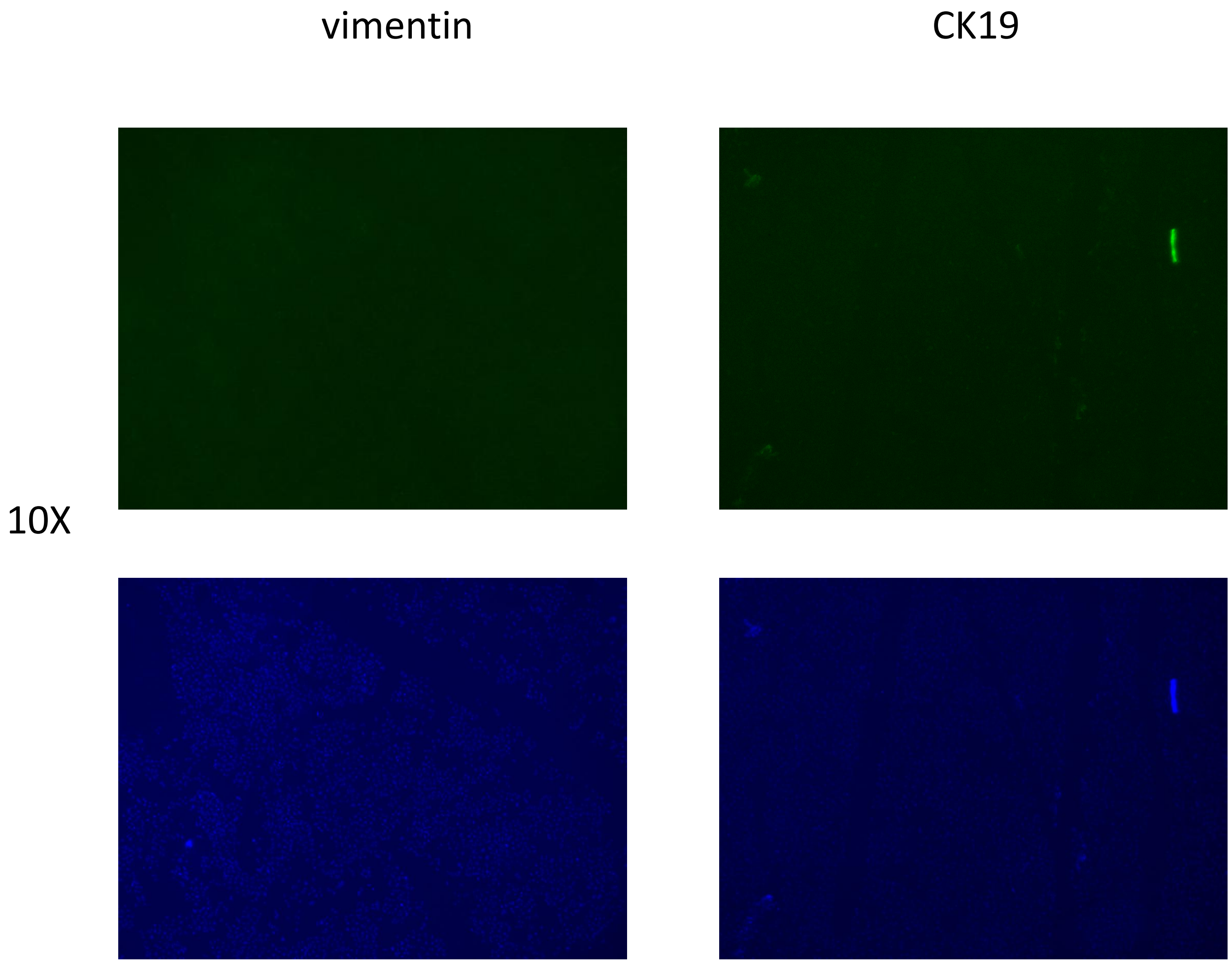

Figure S5. Negative controls of immunofluorescence staining for A549

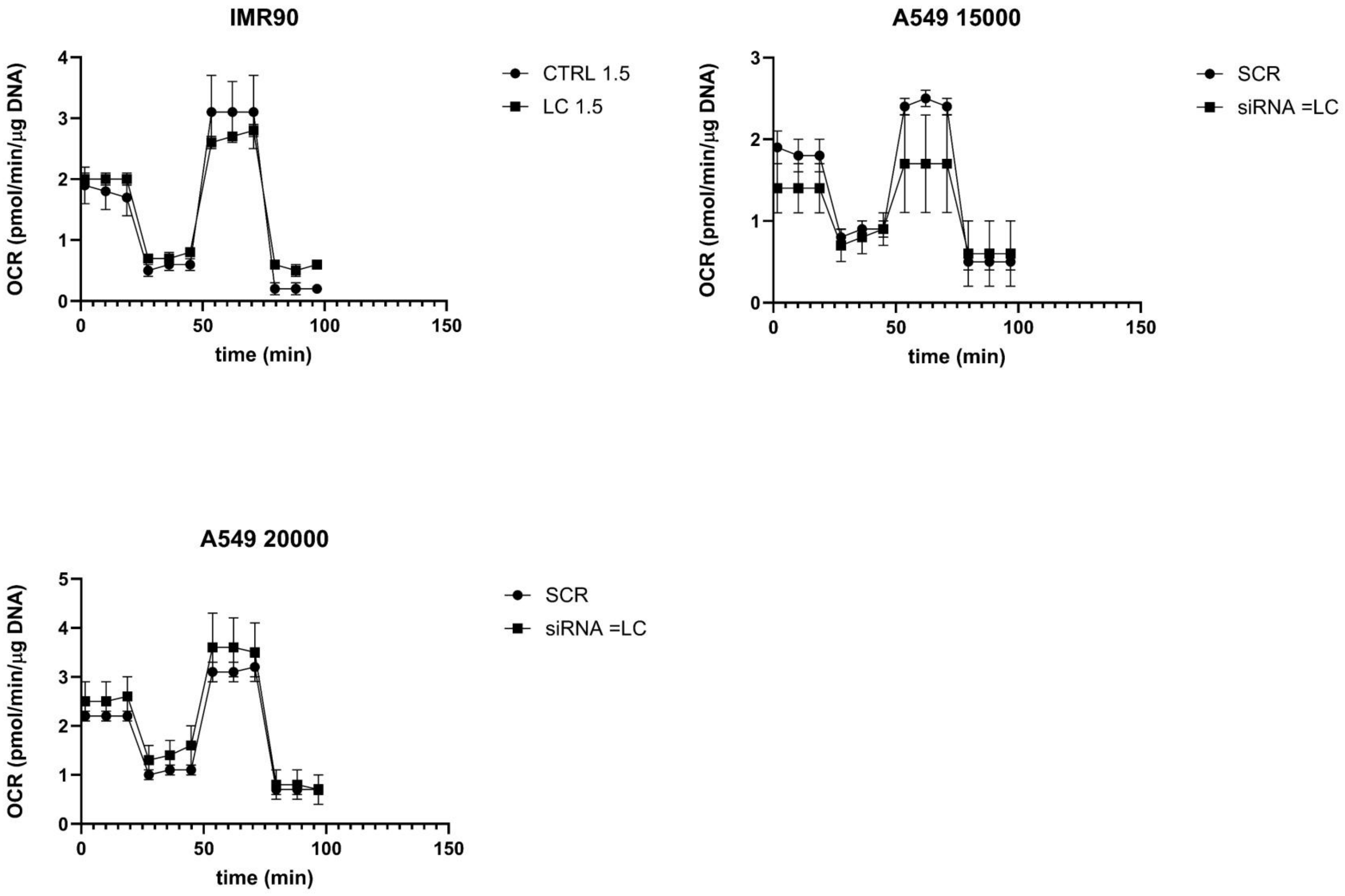

Figure S6. Oxygen consumption rate (OCR) profiles.

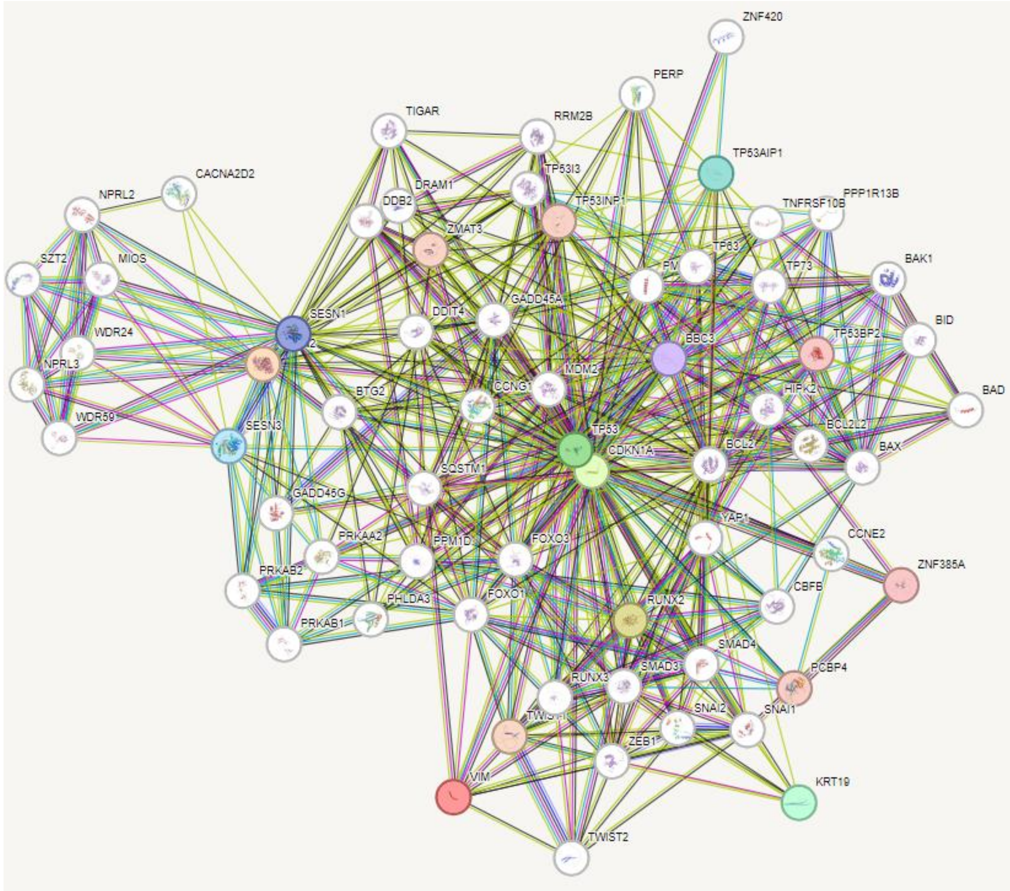

Biological Process (Gene Ontology)

GO-term	description	count in network	strength	signal	false discovery rate
GO:0072331	Signal transduction by p53 class mediator	17 of 93	1.74	4.87	6.96e-21
GO:0072332	Intrinsic apoptotic signaling pathway by p53 class mediator	13 of 56	1.84	4.36	5.99e-17
GO:0008630	Intrinsic apoptotic signaling pathway in response to DNA damage	13 of 75	1.71	3.84	1.09e-15
GO:0097193	Intrinsic apoptotic signaling pathway	18 of 166	1.51	3.83	7.18e-19
GO:0032007	Negative regulation of TOR signaling	11 of 52	1.8	3.63	5.95e-14

(more ...)

Figure S7. Protein interactions analysis
