# Supplementary material for: Extracellular vesicles from long COVID patients promote RUNX2-mediated cellular stress via dysregulated miR-204 and p53 pathway activation
Source: Cell Commun Signal. 2025 Nov 26;23:508. doi: 10.1186/s12964-025-02502-7 (PMC12659154; doi:10.1186/s12964-025-02502-7)
Supplement: Supplementary file 2 — Supplementary Material 2. [file 12964_2025_2502_MOESM2_ESM.docx]

**Table S1.** List of probes analyzed through TaqMan Real-Time PCR

| Gene | Origin | Code |
| --- | --- | --- |
| RUNX2 | Applied Biosystems | Hs1047973_m1 |
| SESN1 | Applied Biosystems | Hs00902782_m1 |
| SOX9 | Applied Biosystems | Hs00165814_m1 |
| PPARG | Applied Biosystems | Hs00608254_m1 |
| β-actin | Applied Biosystems | Hs99999903_m1 |
