# Supplementary material for: Extracellular vesicles from long COVID patients promote RUNX2-mediated cellular stress via dysregulated miR-204 and p53 pathway activation
Source: Cell Commun Signal. 2025 Nov 26;23:508. doi: 10.1186/s12964-025-02502-7 (PMC12659154; doi:10.1186/s12964-025-02502-7)
Supplement: Supplementary file 3 — Supplementary Material 3. [file 12964_2025_2502_MOESM3_ESM.docx]

**Table S2.** List of primers analyzed through SYBR Green Real-Time PCR

| Gene | Origin | Code |
| --- | --- | --- |
| β-actin forward | Invitrogen – Thermo Fisher Scientific | 5’-GAAGGATTCCTATGTGGGCG-3’ |
| β-actin reverse | Invitrogen – Thermo Fisher Scientific | 5’-GGTCTCAAACATGATCTGGGT-3’ |
| SESN2 forward | Invitrogen – Thermo Fisher Scientific | 5’-AGATGGAGAGCCGCTTTGAGCT-3’ |
| SESN2 reverse | Invitrogen – Thermo Fisher Scientific | 5’-CCGAGTGAAGTCCTCATATCCG-3’ |
| P21 forward | Invitrogen – Thermo Fisher Scientific | 5’-GACTCTCAGGGTCGAAA-3’ |
| P21 reverse | Invitrogen – Thermo Fisher Scientific | 5’-TTTGGAGTGGTAGAAATC-3’ |
