# Supplementary material for: Extracellular vesicles from long COVID patients promote RUNX2-mediated cellular stress via dysregulated miR-204 and p53 pathway activation
Source: Cell Commun Signal. 2025 Nov 26;23:508. doi: 10.1186/s12964-025-02502-7 (PMC12659154; doi:10.1186/s12964-025-02502-7)
Supplement: Supplementary file 4 — Supplementary Material 4. [file 12964_2025_2502_MOESM4_ESM.pdf]

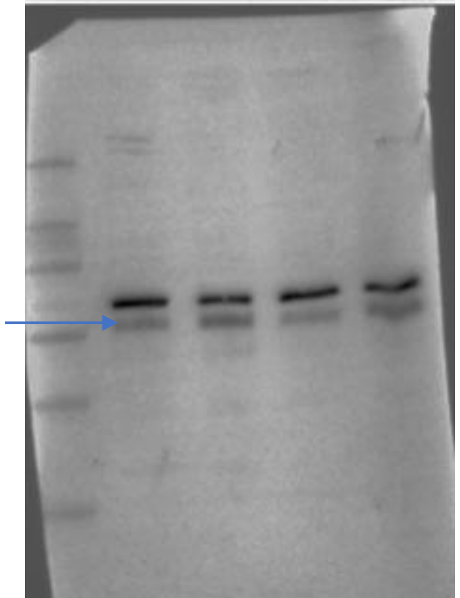

Figure 2F, lane 2 and 3 RUNX2

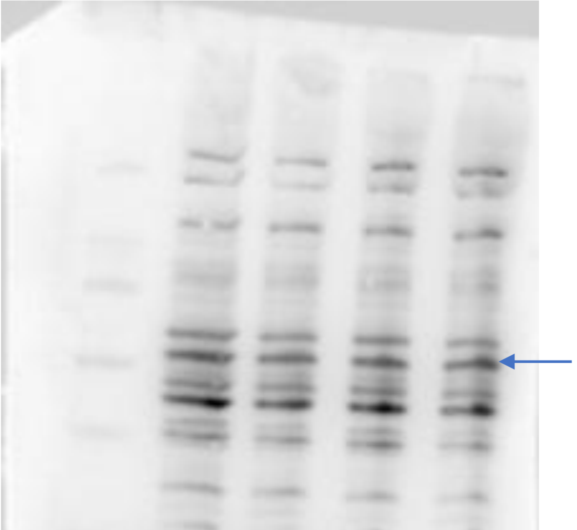

Figure 2F, lane 4 and 5 SESN1

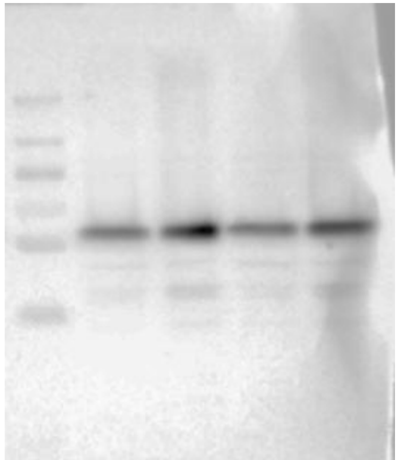

Figure 2F, lane 2 and 3 SESN 2

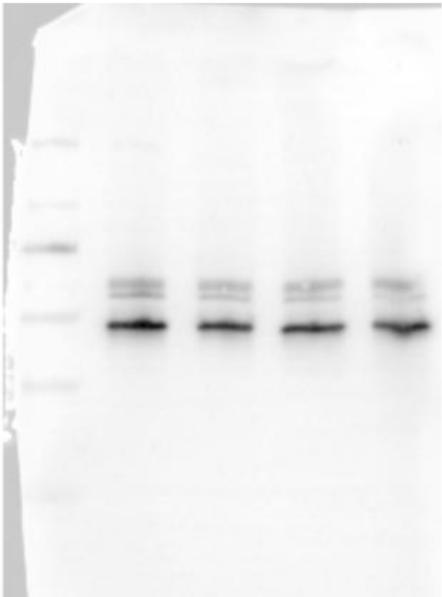

Figure 2F, lane 4 and 5 p53

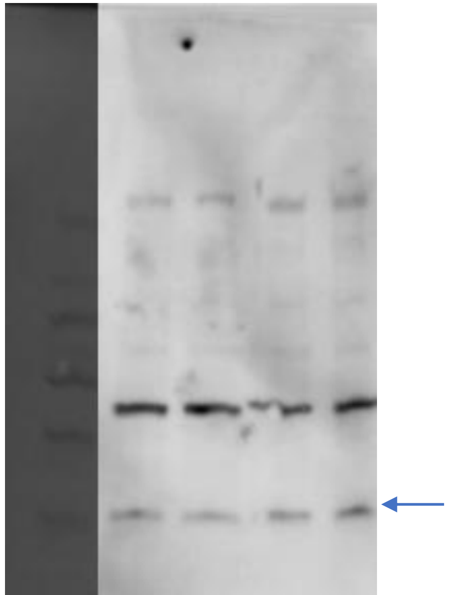

Figure 2F, lane 4 and 5 p21

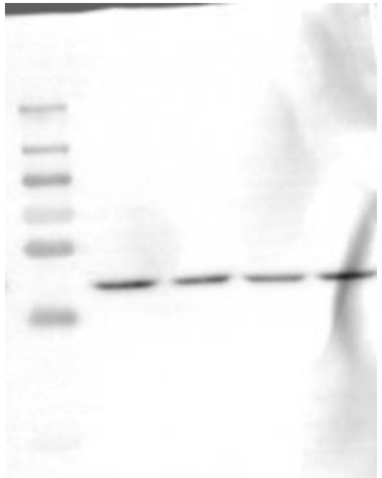

Figure 2F, lane 2 and 3 B actin

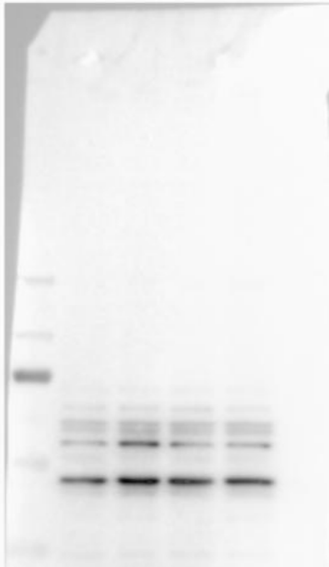

Figure 2G, lane 2 and 3 p53

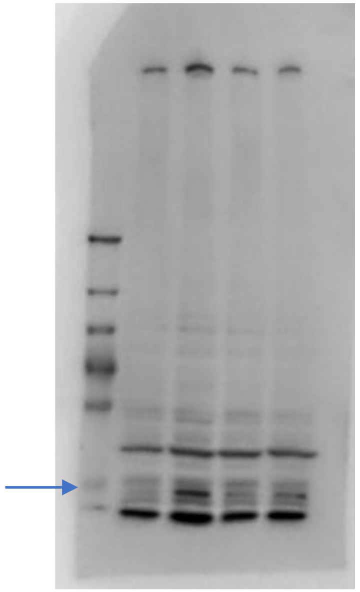

Figure 2G, lane 2 and 3 P-p53

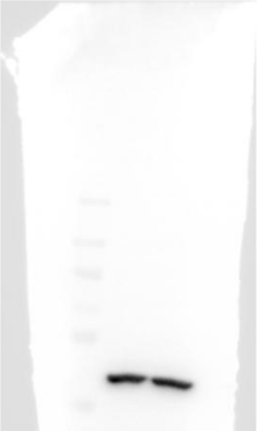

Figure 2G, lane 2 and 3 B actin

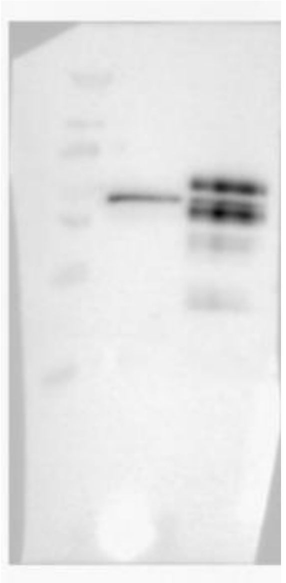
Figure 5B, lane 2 and 3 RUNX2

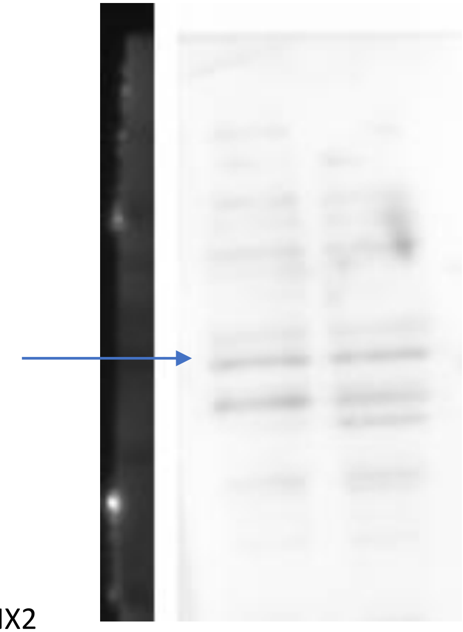
Figure 5B, lane 2 and 3 SESN1

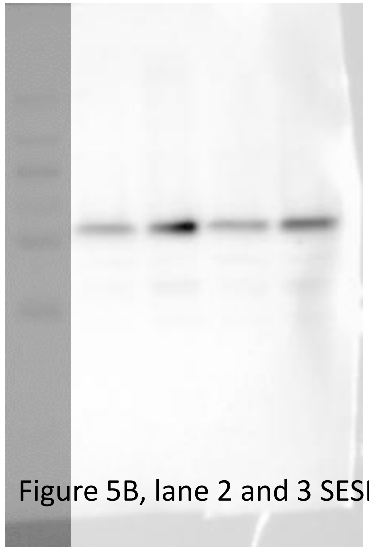
Figure 5B, lane 2 and 3 SESN2

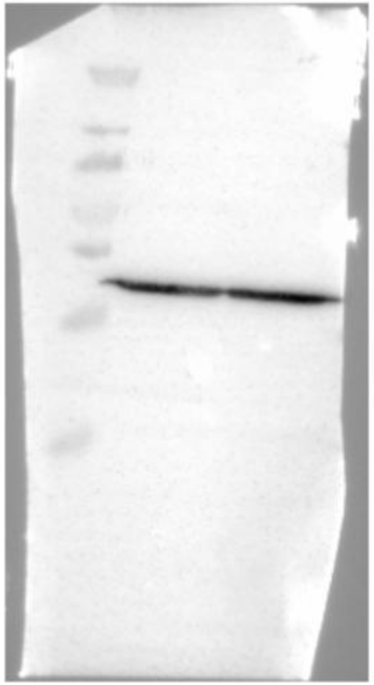
Figure 5, lane 2 and 3 B actin

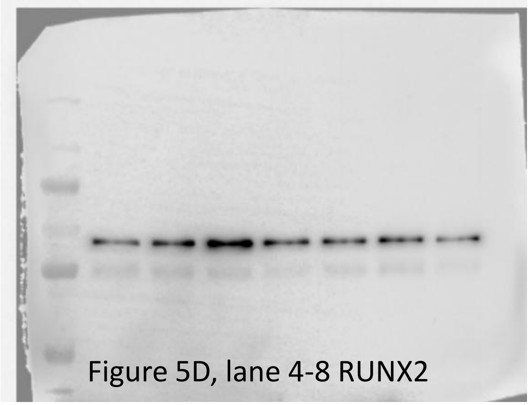
Figure 5D, lane 4-8 RUNX2

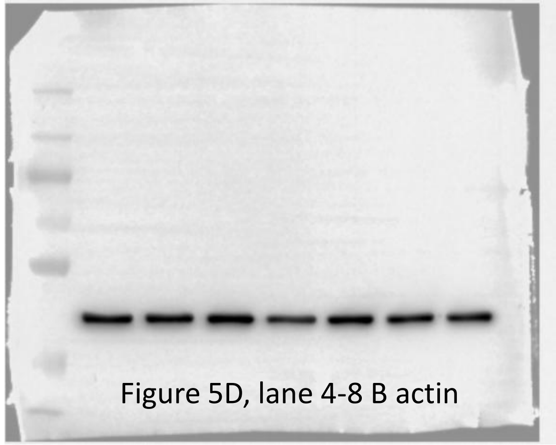
Figure 5D, lane 4-8 B actin

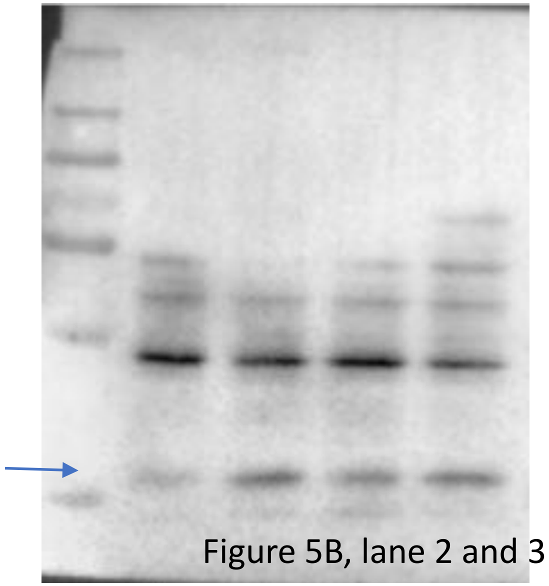
Figure 5B, lane 2 and 3 p21

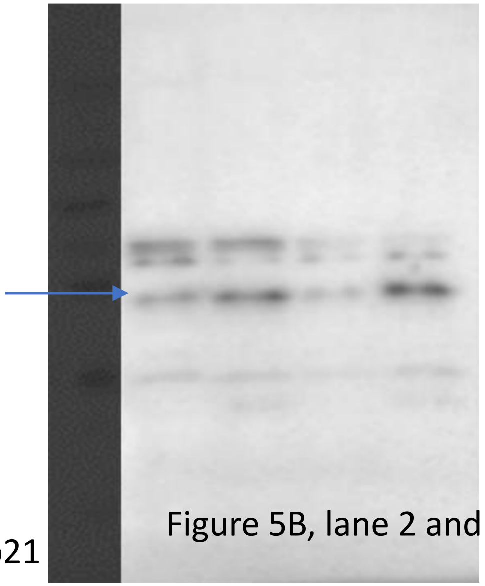
Figure 5B, lane 2 and 3 p53

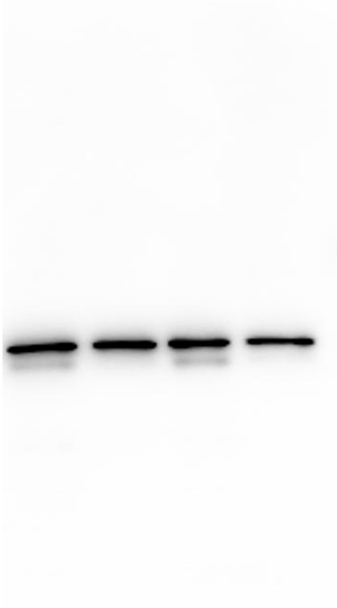

Figure 5C, lane 3 and 4 RUNX2

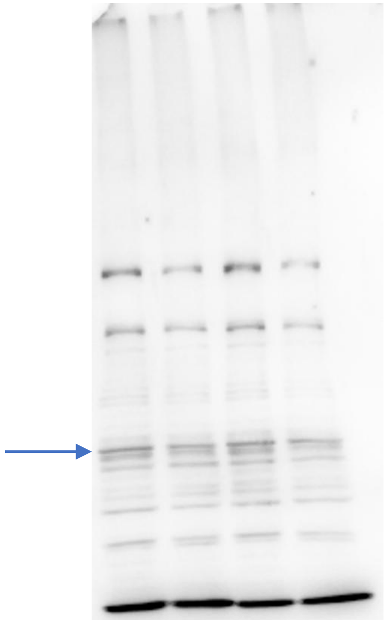

Figure 5C, lane 1 and 2 SESN1

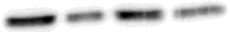

Figure 5C, lane 1 and 2 SESN2

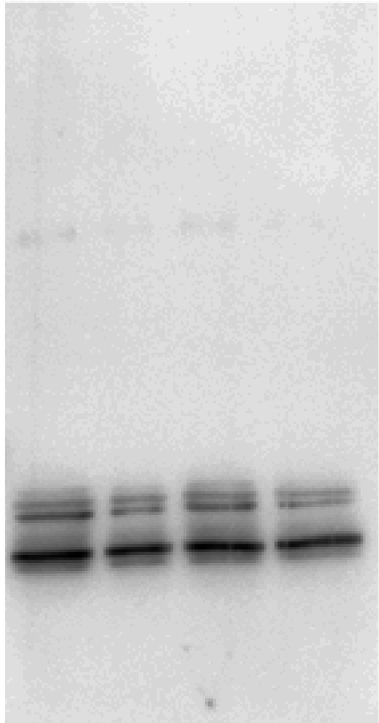

Figure 5C, lane 1 and 2 p53

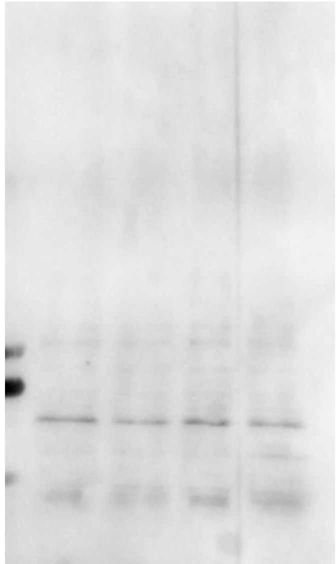

Figure 5C, lane 2 and 3 P-p53

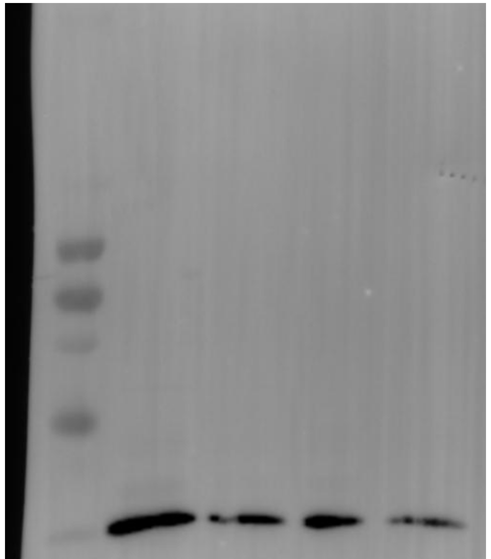

Figure 5C, lane 2 and 3 p21

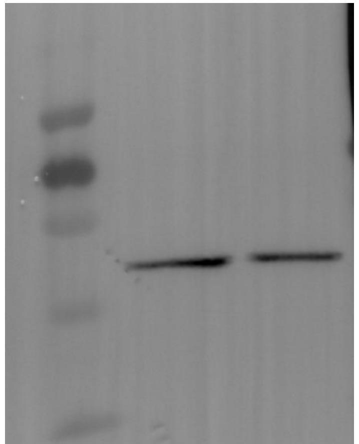

Figure 5C, lane 2 and 3 B Actin

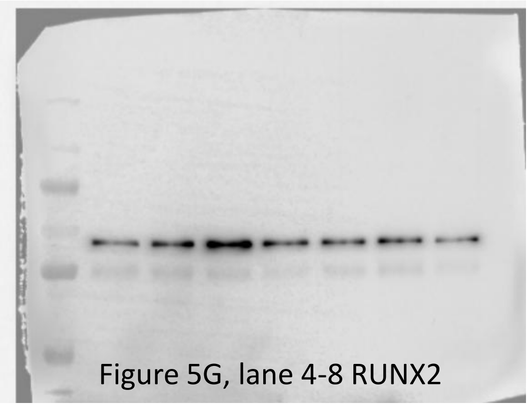
Figure 5G, lane 4-8 RUNX2

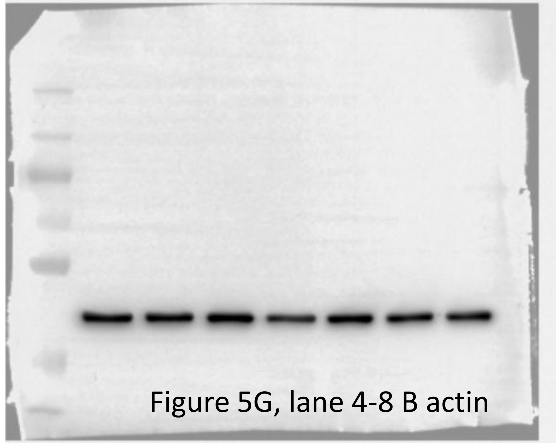
Figure 5G, lane 4-8 B actin

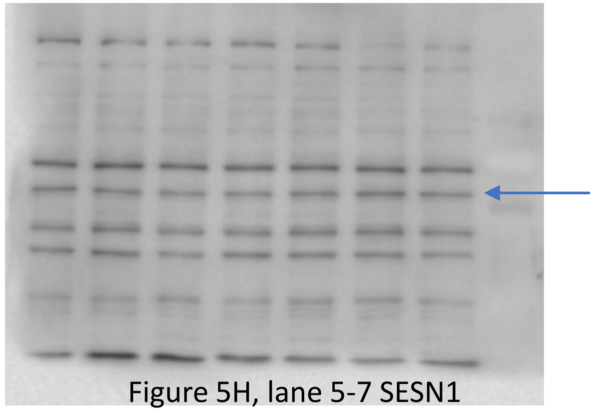
Figure 5H, lane 5-7 SESN1

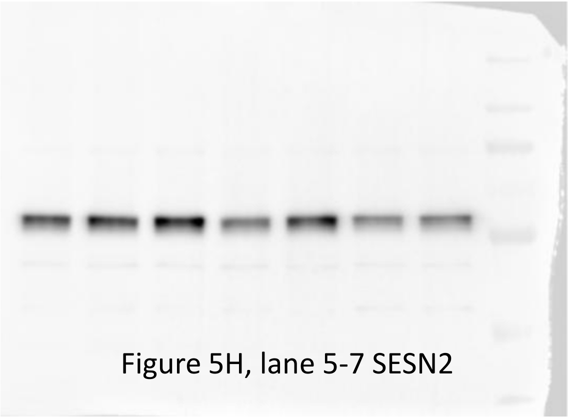
Figure 5H, lane 5-7 SESN2

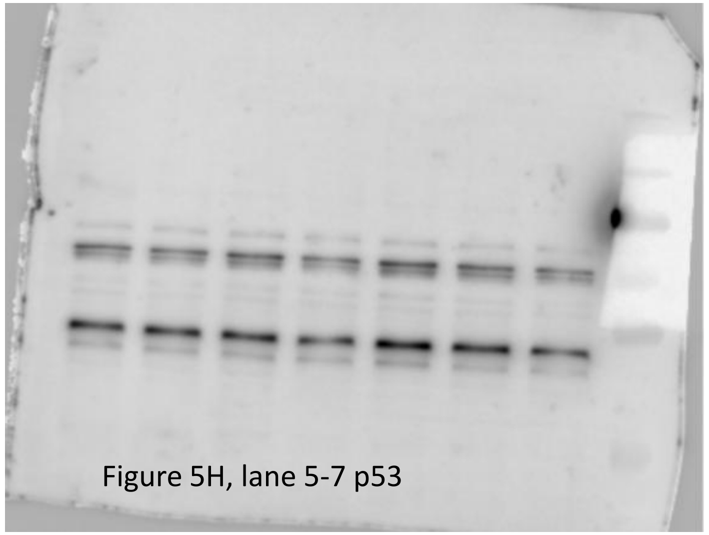
Figure 5H, lane 5-7 p53

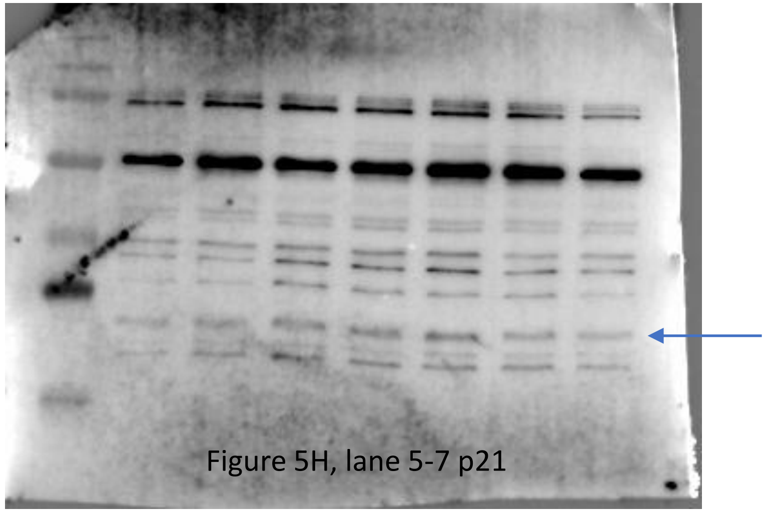
Figure 5H, lane 5-7 p21

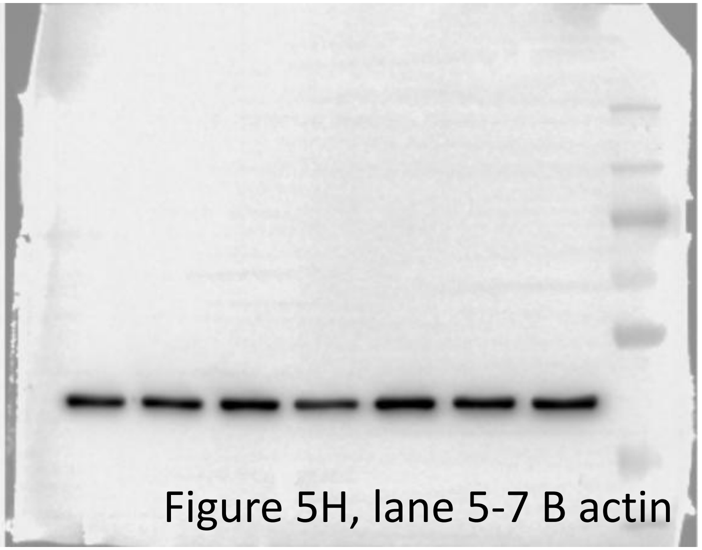
Figure 5H, lane 5-7 B actin

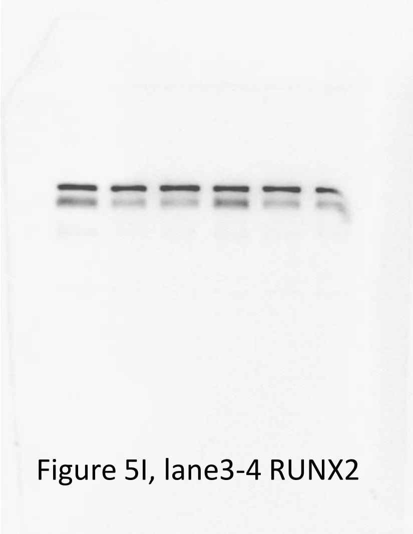

Figure 5I, lane3-4 RUNX2

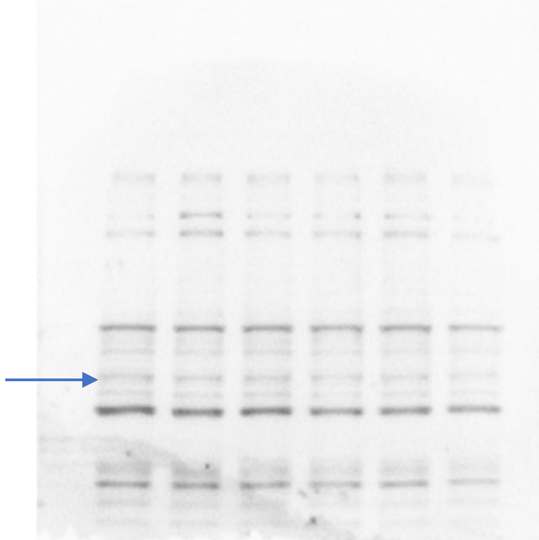

Figure 5I, lane3-4 SESN1

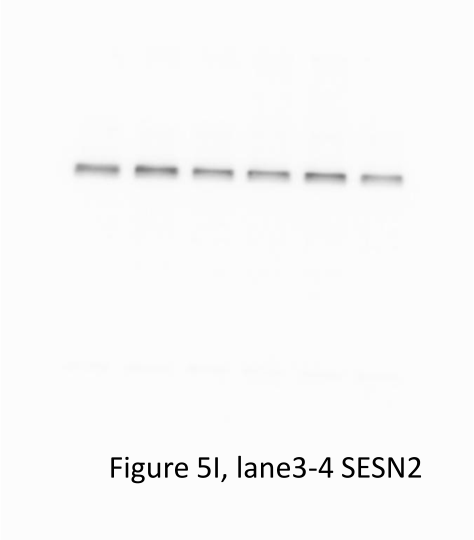

Figure 5I, lane3-4 SESN2

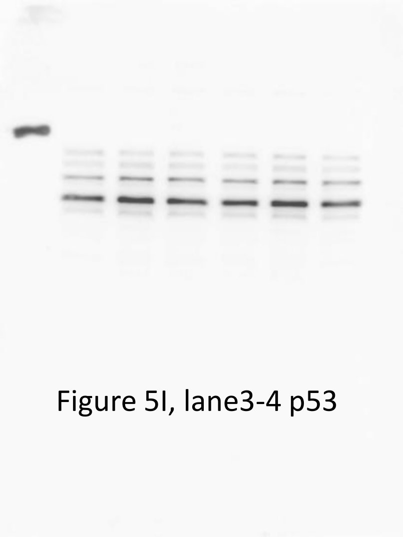

Figure 5I, lane3-4 p53

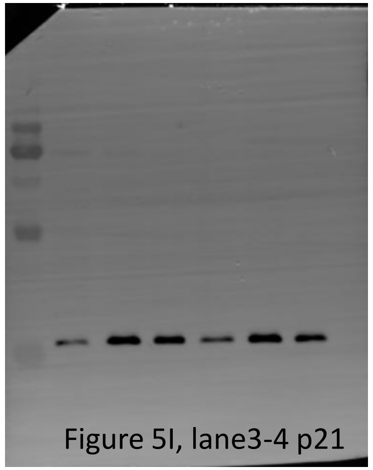

Figure 5I, lane3-4 p21

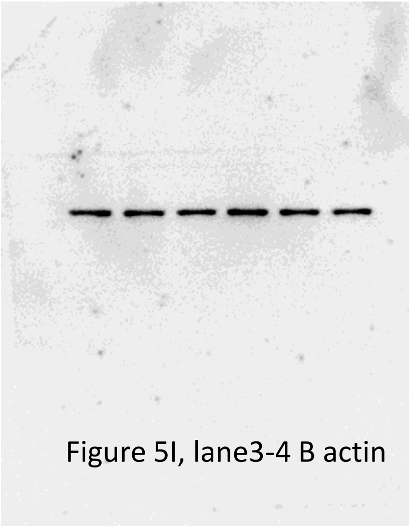

Figure 5I, lane3-4 B actin

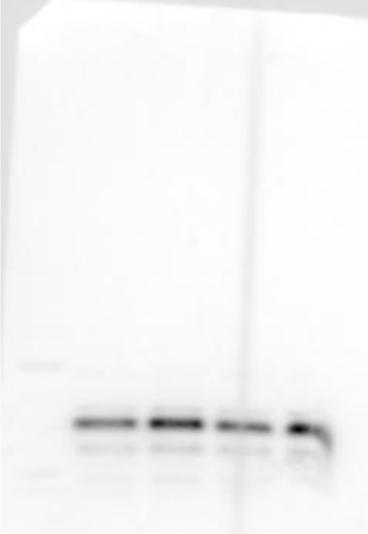

Figure 6A, lane2-3 RUNX2

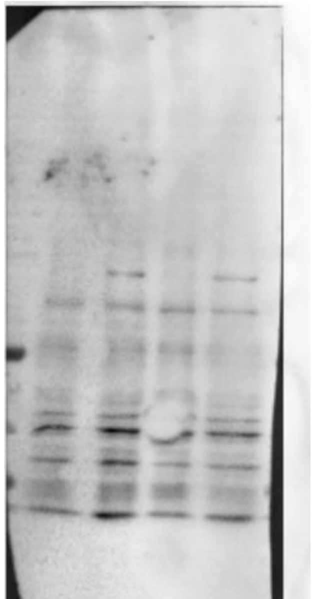

Figure 6A, lane2-3 SESN1

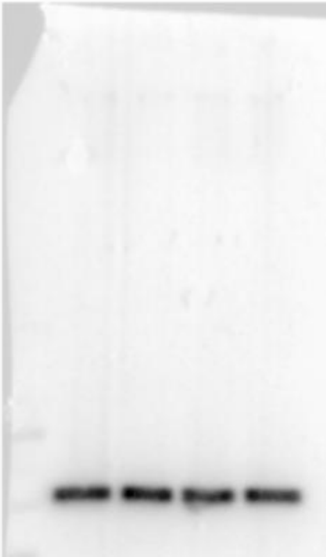

Figure 6A, lane2-3 SESN2

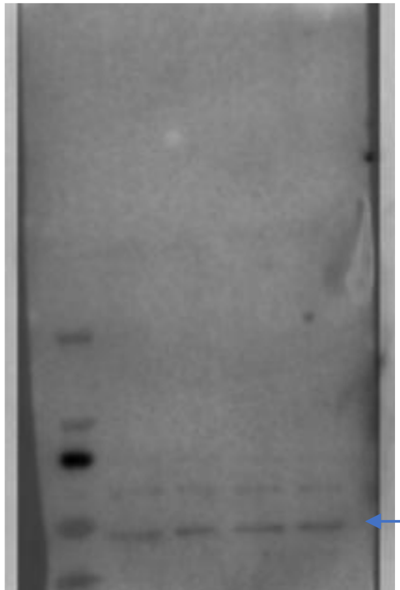

Figure 6A, lane 4-5 p53N2

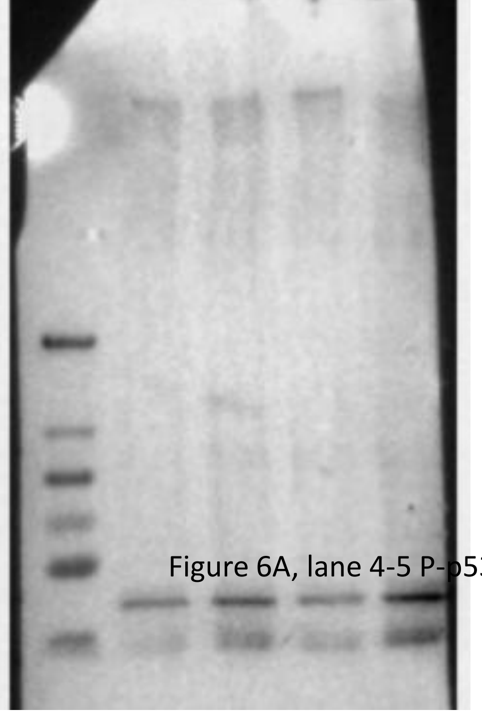

Figure 6A, lane 4-5 P-p53

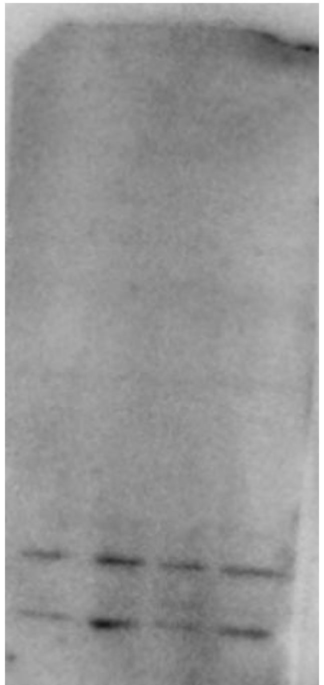

Figure 6A, lane 1-2 p21

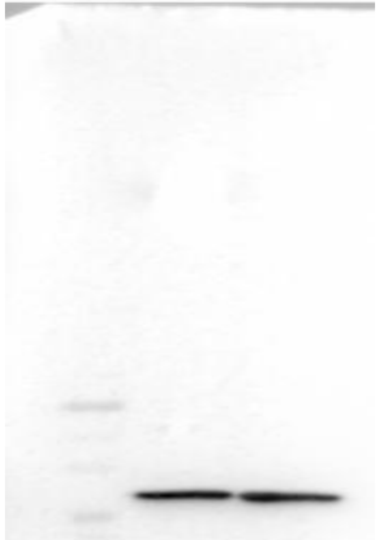

Figure 6A, lane 2-3 B Actin

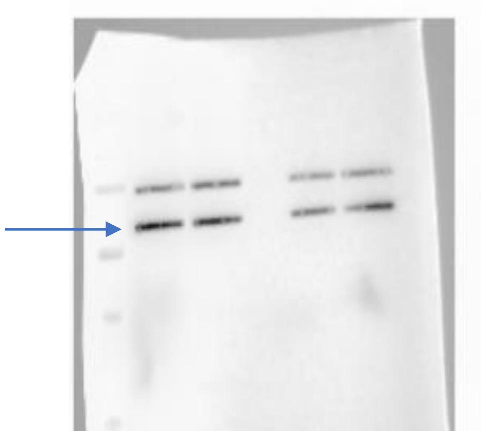
Figure 7C lane 5 and 6 RUNX2

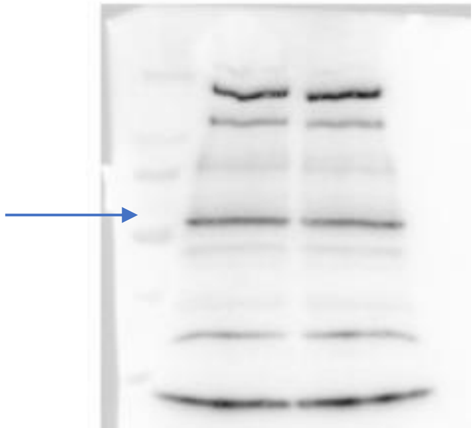
Figure 7C lane 2 and 3 SESN1

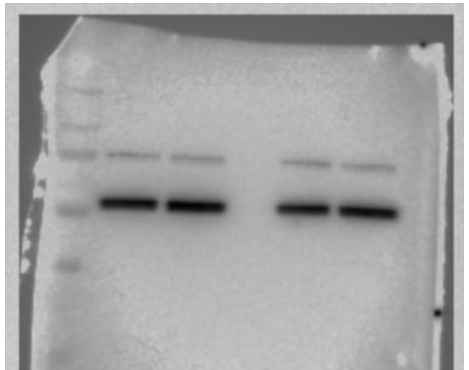
Figure 7C lane 5 and 6 SESN2

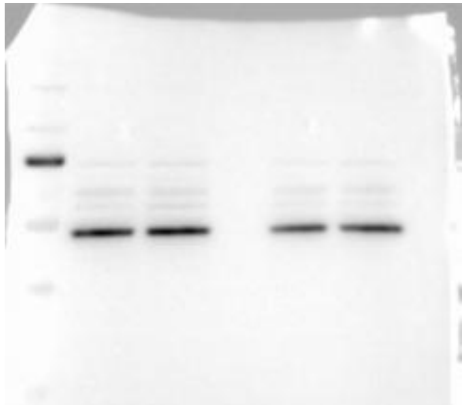
Figure 7C lane 5 and 6 p53

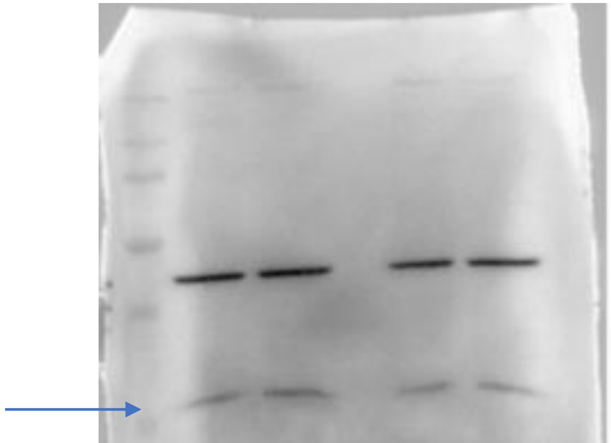
Figure 7C lane 2 and 3 p21

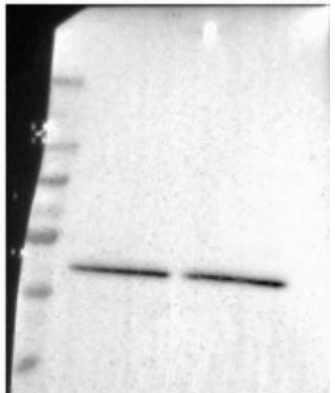
Figure 7C lane 2 and 3 B actin

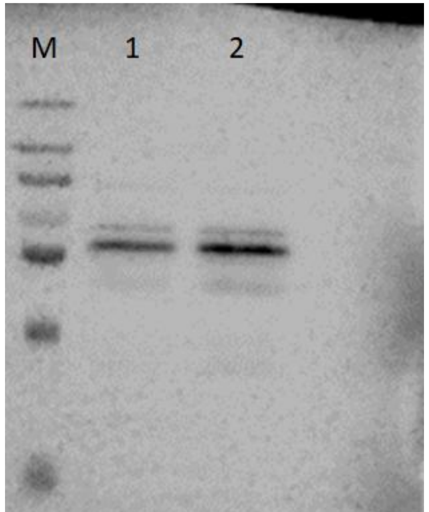

Figure 7F lane 2 and 3 RUNX2

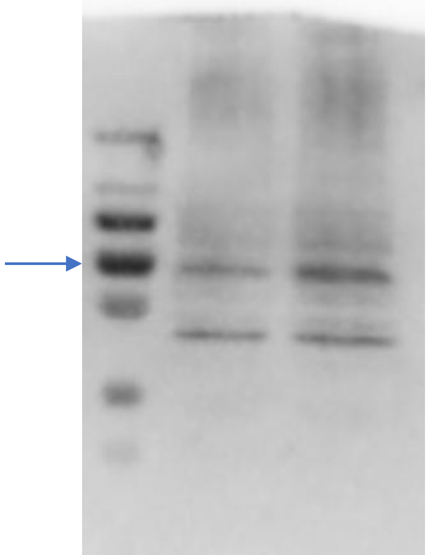

Figure 7F lane 2 and 3 SESN1

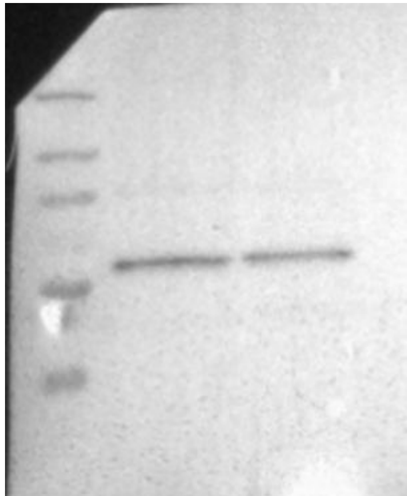

Figure 7F lane 2 and 3 SESN2

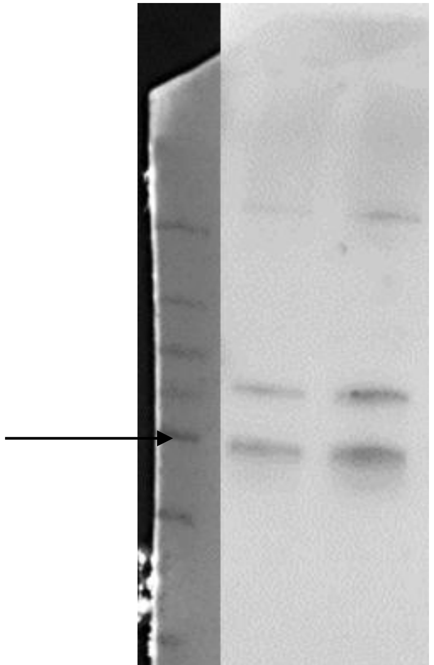

Figure 7F lane 2 and 3 p53

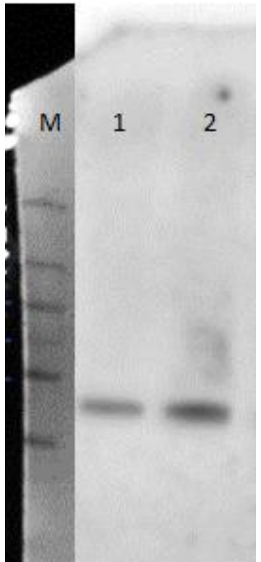

Figure 7F lane 2 and 3 p21

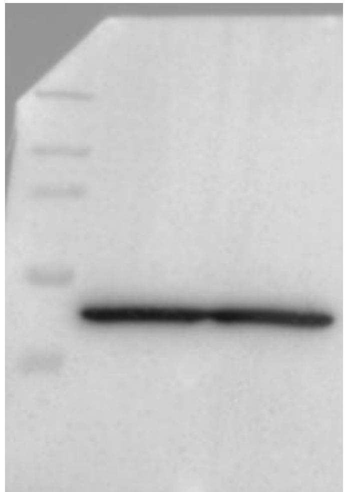

Figure 7F lane 2 and 3 B actin

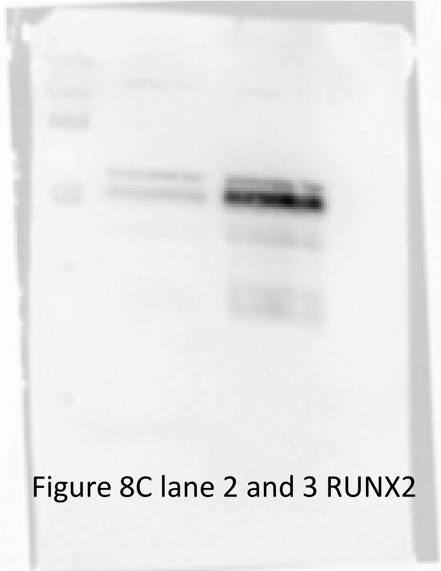

Figure 8C lane 2 and 3 RUNX2

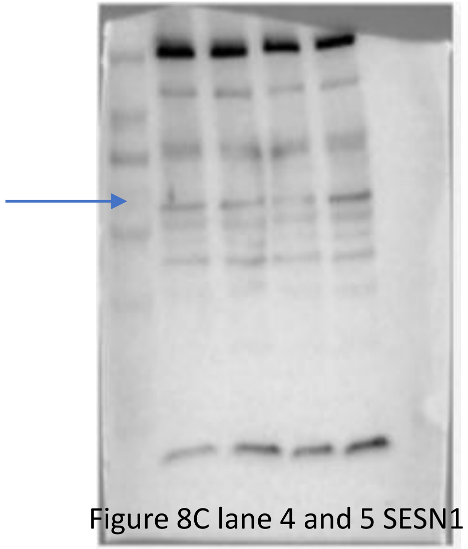

Figure 8C lane 4 and 5 SESN1

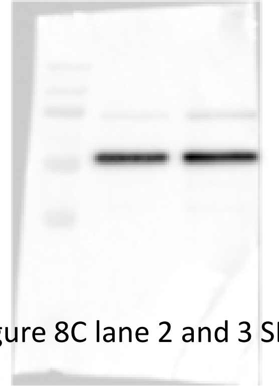

Figure 8C lane 2 and 3 SESN2

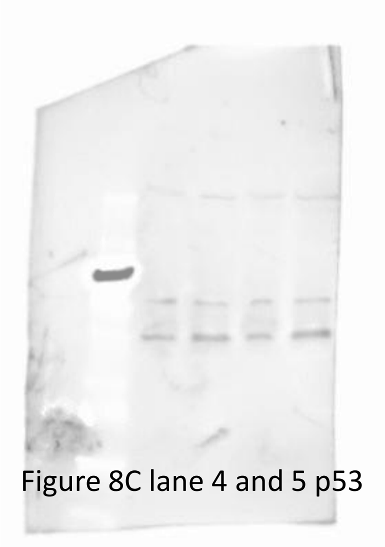

Figure 8C lane 4 and 5 p53

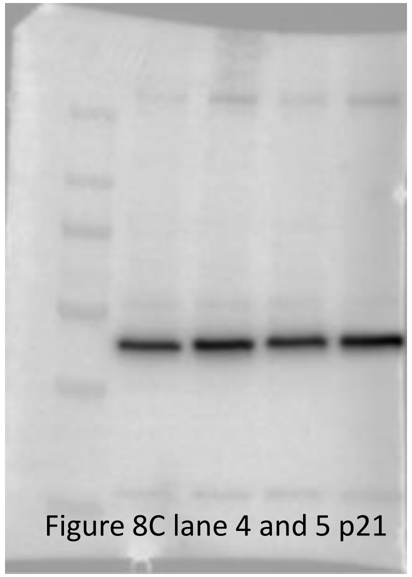

Figure 8C lane 4 and 5 p21

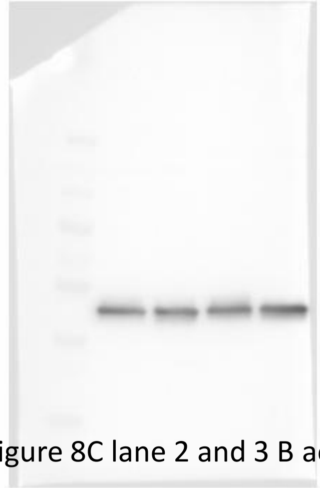

Figure 8C lane 2 and 3 B actin

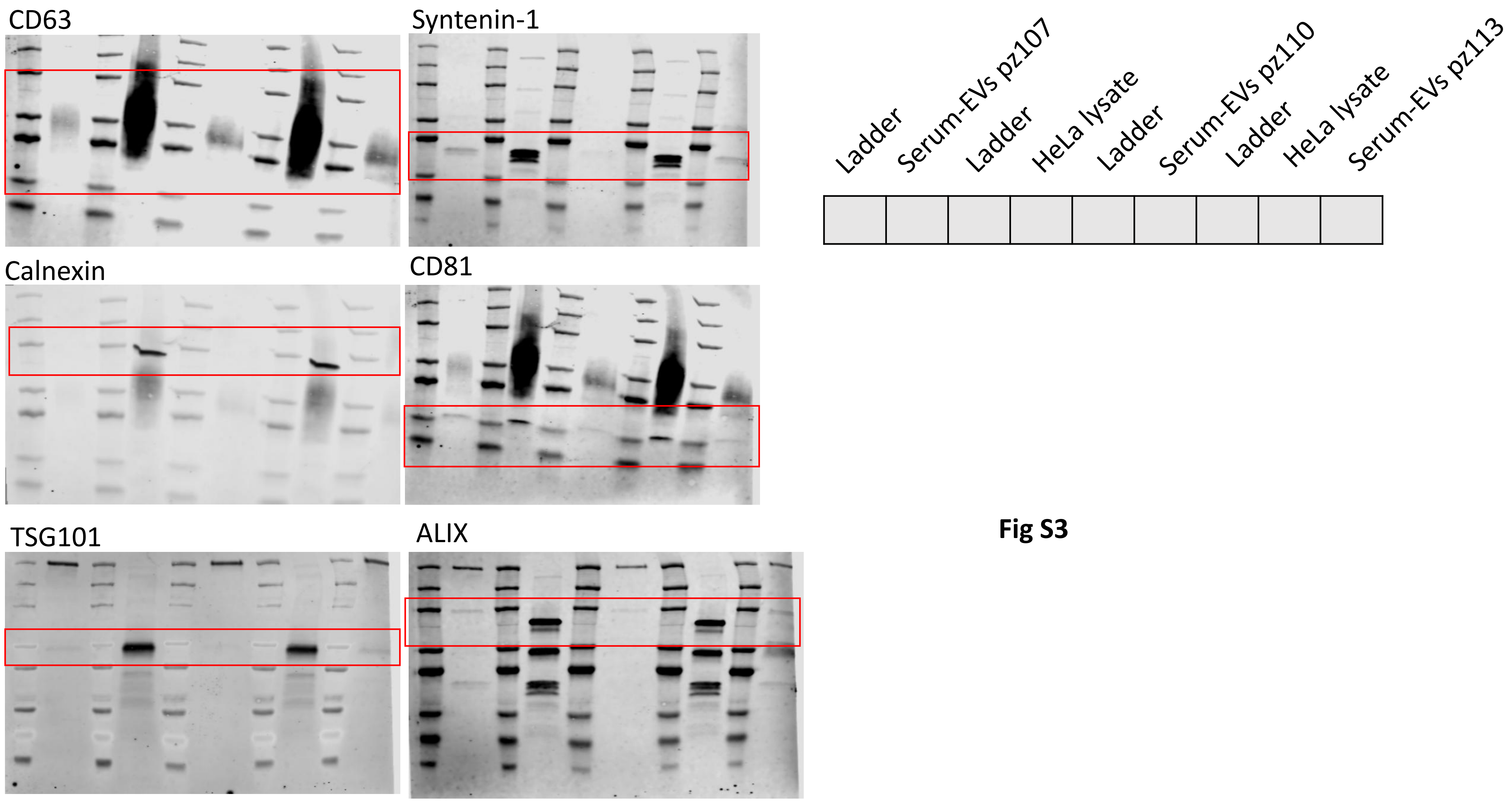
CD63
Syntenin-1
Calnexin
CD81
TSG101
ALIX
Ladder
Serum-EVs pz107
Ladder
HeLa lysate
Ladder
Serum-EVs pz110
Ladder
HeLa lysate
Serum-EVs pz113

Fig S3
